# Cell Dynamics in WOX5-Overexpressing Root Tips: The Impact of Local Auxin Biosynthesis

**DOI:** 10.3389/fpls.2020.560169

**Published:** 2020-10-22

**Authors:** Maria S. Savina, Taras Pasternak, Nadya A. Omelyanchuk, Daria D. Novikova, Klaus Palme, Victoria V. Mironova, Viktoriya V. Lavrekha

**Affiliations:** ^1^Institute of Cytology and Genetics, SB RAS, Novosibirsk, Russia; ^2^Institute of Biology II/Molecular Plant Physiology, Centre for BioSystems Analysis, BIOSS Centre for Biological Signalling Studies University of Freiburg, Freiburg, Germany; ^3^LCTEB, Novosibirsk State University, Novosibirsk, Russia

**Keywords:** RAM, auxin, WOX5, mitotic activity, mathematical model, image analysis, iRoCS toolbox, EdU

## Abstract

Root stem cell niche functioning requires the formation and maintenance of the specific “auxin-rich domain” governed by directional auxin transport and local auxin production. Auxin maximum co-localizes with the WOX5 expression domain in the quiescent center that separates mitotically active proximal and distal root meristems. Here we unravel the interconnected processes happening under WOX5 overexpression by combining *in vivo* experiments and mathematical modeling. We showed that WOX5-induced TAA1-mediated auxin biosynthesis is the cause, whereas auxin accumulation, PIN transporters relocation, and auxin redistribution between proximal and distal root meristems are its subsequent effects that influence the formation of the well-described phenotype with an enlarged root cap. These findings helped us to clarify the role of WOX5, which serves as a local QC-specific regulator that activates biosynthesis of non-cell-autonomous signal auxin to regulate the distal meristem functioning. The mathematical model with WOX5-mediated auxin biosynthesis and auxin-regulated cell growth, division, and detachment reproduces the columella cells dynamics in both wild type and under WOX5 dysregulation.

## Introduction

Plant root apical meristem is a classical stem cell niche where an organizing center (the quiescent center, QC) produces local signals promoting maintenance of the adjacent stem cells (initials). The QC separates distal and proximal portions of the meristem that give rise to the root cap and the rest of the root body, respectively. The QC-produced WOX5 (WUSCHEL-RELATED HOMEOBOX 5) transcription factor provides for maintenance of the columella stem cells (CSCs) in the distal meristem (van den Berg et al., 1997; Sarkar et al., 2007; Pi et al., 2015; Berckmans et al., 2020). In the *wox5* loss-of-function mutant CSCs prematurely differentiate and obtain starch granules, whereas in the 35S:WOX5-GR overexpressing transgene upon dexamethasone (DEX) treatment an increase in the amount of small stem-like cells not undergoing normal differentiation occurs (Sarkar et al., 2007).

Molecular mechanisms of WOX5 action on CSC include direct inhibition of *CYCLING DOF FACTOR 4* (*CDF4*) transcription (Pi et al., 2015) and the CLAVATA3/ESR-RELATED40 (CLE40) – ARABIDOPSIS CRINKLY4 (ACR4)-CLAVATA1 (CLV1)-WOX5 interplay that restricts WOX5 expression domain (Stahl and Simon, 2009; Stahl et al., 2013). Until recently, it was believed that the WOX5 transcription factor mobility that generates the WOX5 gradient from the QC to CSCs is essential for distal meristem maintenance. However, an in-depth analysis of the CLE40-ACR4-CLV1 pathway suggested that WOX5 mobility is not required to inhibit CSC differentiation and WOX5 acts mainly in the QC (Berckmans et al., 2020). Moreover, it was suggested that other non-cell-autonomous regulators originating in the QC exist to maintain the distal meristem together with CLE40-ACR4-CLV1 circuit (Richards et al., 2015; Berckmans et al., 2020). Thus, the WOX5 role in distal meristem functioning needs to be revisited.

A perfect candidate for the non-cell-autonomous morphogenic substance to supplement CLE40-ACR4-CLV1 circuit in the regulation of distal meristem functioning is the plant hormone auxin. The auxin concentration maximum in the QC is required for maintenance of the adjacent stem cells, including CSCs, and for differentiation of CSC daughters (CSCDs) (Sabatini et al., 1999; Blilou et al., 2005). The distal meristems of several auxin response mutants resemble those developed under disturbed WOX5 expression. The double mutant on AUXIN RESPONSE FACTORs acting as repressors (*arf10-2 arf16-2*) has an extra number of CSCDs with blocked differentiation, where only distal ones have starch granules (Wang et al., 2005; Ding and Friml, 2010). The gain-of-function *axr3* mutant with a stable version of auxin response inhibitor protein INDOLE-3-ACETIC ACID INDUCIBLE 17 (IAA17)/AUXIN RESISTANT 3 (AXR3) has reduced both differentiation and division potential of CSCs and their daughters (Sabatini et al., 1999).

Tight interconnection was proposed between auxin and WOX5 pathways. By mutant analysis, Ding and Friml (2010) suggested that auxin acts upstream of WOX5. Tian et al. (2014b) proposed a feedback mechanism wherein WOX5 serves as a downstream negative target of an auxin response pathway, but it activates tryptophan-dependent auxin-biosynthesis. It was shown by qPCR that *YUCCA1* (*YUC1*), but not *TRYPTOPHAN AMINOTRANSFERASE OF ARABIDOPSIS1* (*TAA1*) is activated by DEX in *35S:WOX5-GR* seedlings (Tian et al., 2014b). Also, auxin response pattern visualized by DR5 reporters, but not the polarity of auxin transport traced by PIN1, PIN4, and PIN2 immunolabeling, were affected in these experiments. Here we revisited the downstream effect of WOX5 on auxin transport, synthesis, and signaling in the distal meristem in greater detail, at a cellular resolution, and with short-term DEX incubations.

A major role of WOX5 in the distal meristem has been proposed when phenotyping the *35S:WOX5-GR* line under DEX treatment. It was suggested that WOX5 represses differentiation in the columella (Sarkar et al., 2007), and its overexpression leads to dedifferentiation of the columella cells into stem-cell-like (Pi et al., 2015). And that WOX5 acts downstream of auxin distribution because auxin treatment does not rescue strong inhibition of DSC differentiation in *35S:WOX5-GR* (Ding and Friml, 2010). As all these conclusions became under question by the recent findings of only the local QC-specific role of WOX5 (Berckmans et al., 2020), we perform reannotation of WOX5 overexpression phenotype and auxin distribution there.

As a result, we found that WOX5-mediated auxin biosynthesis is sufficient to explain the phenotypes of WOX5 gain- and loss-of-function mutants. Moreover, WOX5 downstream effect on auxin in the QC is essential to mediate distal meristem functioning in wild type. These results suggest that one of the main WOX5 roles in the distal meristem is the tuning of the auxin pattern.

## Materials and Methods

### Plant Materials

The following *Arabidopsis thaliana* (L.) lines were used in the experiments: Col-0; *wox5-1* (Sarkar et al., 2007), *35S:WOX5-GR* (Sarkar et al., 2007), TAA1:TAA1-GFP, DR5:GFP and WOX5:GFP (Blilou et al., 2005). TAA1:TAA1-GFP, DR5:GFP, and WOX5:GFP were introduced into the *wox5-1* and *35S:WOX5-GR* backgrounds.

### Growth Conditions and Treatments

Seeds were surface-sterilized and sown on the square Petri dishes containing 1/2 MS medium with 1% (wt/vol) sucrose and 1% (wt/vol) agar (Roth). The dishes were kept at room temperature for 4 h before transfer to 4°C for 12 h. Dishes were then placed at 22°C under long-day (16/8 h light/dark) conditions for the next 96 h with light intensity 80 μmol/m2/sec.

For anatomical studies, four dag seedlings (*35S:WOX5-GR*) were transferred to the new plates containing 1/2 MS medium supplemented with 15 μM DEX (*N* = 10). For the study of the L-kynurenine (K8625 Sigma-Aldrich) effect, three dag *35S:WOX5-GR* seedlings were transferred to the liquid 1/2 MS media containing 15 μM DEX with and without L-kynurenine (0.2 μM) for 48 h. For the control, we used three dag *35S:WOX5-GR* seedlings transferred to the liquid 1/2 MS media without DEX. EdU was added to both media for the last 4 h of DEX treatment.

For fluorescence measuring, three dag seedlings of *35S:WOX5-GR* TAA1:TAA1-GFP crossed line were treated for 6, 24, and 48 h with 15 μM DEX in liquid 1/2 MS medium (*N* = 20).

In all the studies we used five dag *wox5-1* seedlings grown on the solid 1/2 MS media.

### Whole-Mount *in situ* Immunolocalization

Immunolocalization was performed according to a whole-mount *in situ* protocol (Pasternak et al., 2015). Affinity purified primary anti-PIN1 (mouse, clone 7E7F, was diluted 1:40), anti-PIN2 (guinea pig, clone 192, 1:400 dilution); anti-PIN4 (rabbit, clone 9105, 1:400 dilution), anti-PIN7 (mouse, clone A875, 1:40 dilution), and anti-GFP (rabbit, cat No, A-21311, 1:200 dilution) were used. The secondary Alexa-488/Alexa 555 conjugated anti-mouse, anti-rabbit and anti-guinea pig antibodies were diluted 1:400^1^. Seedlings (*N* = 10) were co-stained with DAPI and mounted on the microscopic slide with 120 μm spacer to prevent the root damage.

### Microscopy and Image Processing

We used the LSM 510 META NLO confocal laser scanning microscope. DAPI/EdU signals were recorded at an excitation wavelength of 740 nm (2-P laser) and 488 nm laser with a C-Apochromat 25×/1.2 glycerol W corrected UV-VIS-IR objective (according to Pasternak et al., 2015).

Confocal images were converted to hdf5 format using the LOCI plugin for ImageJ^2^. Representative roots were chosen for detailed annotation with the iRoCS Toolbox (Schmidt et al., 2014). The DAPI and EdU channel images were processed as reported in Lavrekha et al. (2020) (Supplementary Table 1).

Root lengths and the intensity of fluorescence signals were measured using ImageJ (see text footnote 2). Statistical analysis for the DEX-induced changes in root length (Supplementary Figure 7) and GFP fluorescence (Figure 3) was done using Welch’s *t*-test (*N* = 20).

ANOVA statistical testing with Tukey *post hoc* test (CI 95%, *N* = 20) has been applied to estimate the significance.

### Real Time PCR

Arabidopsis three dag plants were treated with 15 μM DEX for 0, 6, and 24 h and with 15 μM DEX 0.2 μM L-kynurenine for 24 h in liquid 1/2 MS media. The root tips (3–4 mm length) of treated plants were cut and frozen in liquid nitrogen. RNA was isolated with TRIzol (Invitrogen) and RNAeasy kit (Qiagen). cDNA was synthesized from 1 μg total RNA with iScript cDNA Synthesis kit (Biorad). Real time PCR was measured on CFX96 RT-PCR detection system using EvaGreen kit (Sintol). Each reaction was performed in three biological repeats. EEFalpha4 was used as a reference gene. All the primers used for qRT-PCR are listed in Supplementary Table 2.

### Mathematical Modeling

#### 2D Model of Auxin Distribution Within the Root Apical Meristem

We used the two-dimensional dual-mechanism mathematical model described in Mironova et al. (2012), Hong et al. (2017)). The model in ODE is implemented in Matlab software and considers the following processes: auxin synthesis, auxin degradation, auxin diffusion, and PIN-mediated active transport, and auxin-dependent expression of PINs. The rectangular cell layout consists of 25 rows (*i* = 1…25) and 10 columns (*j* = 1…10) (see Supplementary Material for the detail). The first row corresponds to the root end, the 25^th^ row to the last cell of the meristem. In the cell layout, there are five tissues: epidermis (*j* = 1, 10), cortex (*j* = 2, 9), endodermis (*j* = 3, 8), and stele (*j* = 4, 5, 6, 7) that differed by expression of PIN auxin transporters (PINse, PINce, and PINnp). Stele and endodermis are able to express PINse with rootward and lateral polarity. Cortex and epidermis cells are able to express PINce, cortex with rootward and lateral polarities, epidermis with shootward and lateral polarities. Potentially every cell of the layout might express PINnp, which transports auxin non-polarly. The rates of PINse, PINce, and PINnp synthesis in a cell depend on the internal auxin level; the rates of PINse and PINce degradation depend on internal auxin levels as well (see details in Supplementary Material).

#### 1D Model of Auxin-Regulated Cell Dynamics in Columella

The hybrid one-dimensional computational model with cell growth, division, and detachment are described by ODE and logical functions in the Matlab software. Auxin dynamics in ODE is represented as TAA1-dependent auxin biosynthesis, PINnp-mediated auxin active transport; passive transport, and degradation. The model depicts the cell dynamics in the distal meristem only, namely QC, CSC, and all their descendants (see Supplementary Material for details).

The model calculation is iterative. After 100 steps of ODE calculation for continuous processes (synthesis, degradation, and growth), the model checks if the condition for the discrete event (such as cell division or detachment) is fulfilled for any of the cells. If yes, the model rewrites the system of ODEs for the modified cell ensemble. To define the cell state in the model (Quiescent, Stem, Differentiation, and Detachment) we used three auxin concentration thresholds (*s*_QC_, *s*_CSC_, and *s*_D_), analogously to Dubreuil et al. (2018).

## Results

### Cell Dynamics in the Distal Meristem Upon Induction of WOX5 Overexpression

**FIGURE 1:**
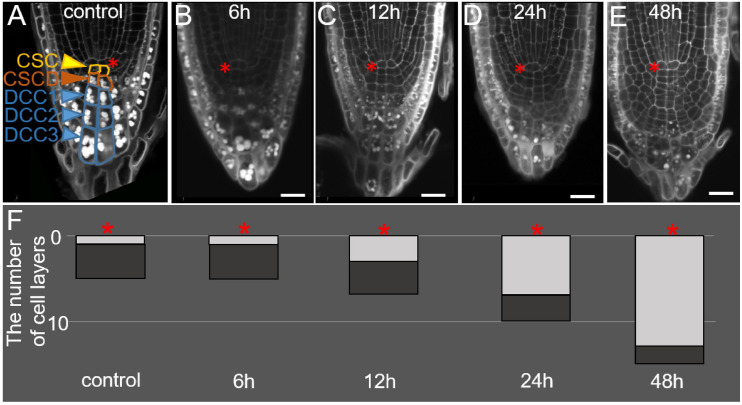
Changes in the root tip structure during DEX incubation in the *35S:WOX5-GR* line. **(A–E)** The root tip anatomy of *35S:WOX5-GR* root tips after 0 h **(A)**, 6 h **(B)**, 12 h **(C)**, 24 h **(D)**, and 48 h **(E)** of DEX treatment. *N* = 10. CSC, columella stem cells; CSCD, columella stem cell daughters; DCC, differentiated columella cells. **(F)** The changes in the number of layers for stem-like cells (light gray) and DCC cells (dark gray) in columella is represented. The QC location is marked by the red asterisks. The bar scale – 20 μm.

**FIGURE 2:**
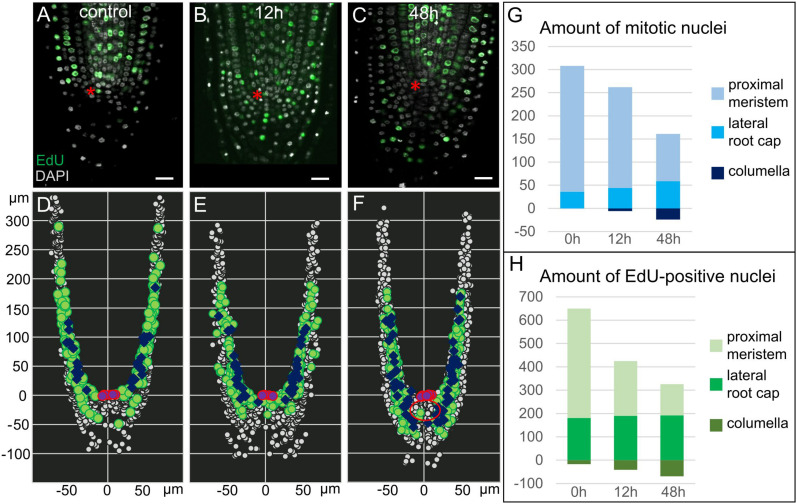
Proliferation activity in *35S:WOX5-GR* root tips. **(A–F)** Distribution of mitotic, S-phase, and interphase nuclei in *35S:WOX5-GR* root tips at 0 **(A,D)**, 12 **(B,E)**, and 48 h **(C,F)** of DEX exposure. **(A–C)** Confocal images of EdU/DAPI labeled root tips. EdU-positive green color marks nuclei undergoing S-phase, highly dense white color marks mitotic nuclei, shallow light gray marks interphase nuclei. The bar scale – 20 μm. The QC location is marked by the red asterisks. **(D–F)** 3D maps built upon **(A–C)** with iRoCs Toolbox (Schmidt et al., 2014). Nuclei are presented as gray dots. EdU-marked cells and mitoses are displayed as green circles and dark blue rhombuses, respectively, interphase nuclei are small dots. QC cells are shown by the magenta circles. **(G,H)** Quantitative analysis of 3D maps for mitotic **(G)** and EdU-positive nuclei **(H)** distribution in the *35S:WOX5-GR* root tips at 0, 12, and 48 h of DEX exposure. The full data is given in Supplementary Table 1.

To clarify the WOX5 role in the distal meristem functioning we performed 3D annotation of *35S:WOX5-GR* root tips under DEX exposure (0, 6, 12, 24, and 48 h). The root tips were labeled to visualize either cell walls and starch grains (Figures 1A–E) or S- and M-phases of the cell cycle (Figures 2A–C) (see “Materials and Methods”). 3D confocal images were analyzed in iRoCs Toolbox (Schmidt et al., 2014) to build the root tip computer models and to study the distribution of different cell types quantitatively (Figures 2D–F). In accordance with the earlier works (Sarkar et al., 2007; Ding and Friml, 2010; Tian et al., 2014a; Pi et al., 2015), we observed that DEX-inducible *WOX5* gene activation leads to the proliferation of stem-like cells in the columella and lateral root cap and a decrease in the number of differentiated starch-containing cells in time. The 3D root tip models helped us to understand the source of the stem-like cells and the destiny of differentiated columella cells (DCC).

During the first six hours of DEX incubation, the columella structure remains similar to mock-treated plants (Figures 1A,B,F). In the next 6 h, the overall number of columella cell layers increased from five to seven (Figures 1A,C,F), whereas the number of layers containing starch granules and corresponding to DCCs did not change (Figures 1A–C,F). An increase in the columella length at 12 h after DEX treatment happened due to precocious divisions of CSCs and the generation of two additional layers of small stem-like cells. After 24 h of DEX treatment and detachment of one root cap layer, the lower part of the columella possessed three DCC layers with starch granules, whereas the upper columella part became composed of seven layers of small stem-cell-like cells, not containing starch granules (Figures 1D,F). This tendency persisted after 48 h of DEX treatment, DCC layers differentiated before the DEX treatment onset were detached leaving in columella novel 10–12 layers generated from the cells being CSCs or CSCDs when the DEX incubation started (Figures 1E,F).

To study proliferation activity upon WOX5 induction in a greater resolution, we monitored the distribution of nuclei in different cell cycle phases by combining EdU and DAPI labeling (see “Materials and Methods”; Figures 2A–C). Using iRoCs Toolbox we built 3D maps of nuclei distribution within the root tips in *35S:WOX5-GR* after DEX incubation for 0, 12, and 48 h and quantified with their help the density of mitotic, interphase and S-phase nuclei over the root tip tissues (Figures 2D–F, Supplementary Dataset 1, and Supplementary Figure 1). From generated 3D maps (Figures 2D–F, Supplementary Dataset 1) it followed that the mitotic and S-phase signals were gradually increased in time in the root cap of the *35S:WOX5-GR* plants. In turn, in the proximal meristem, we observed a decrease in proliferation activity (Figures 2G,H and Supplementary Table 1). Localization of mitotic and S-phase events significantly differed from the control after 48 h of DEX incubation: the majority of the cells in the S- and M-phases in control plants have been localized in the lateral root cap at the level of the QC, while upon DEX induction cell cycle progression has been mainly detected in the upper columella (Figures 2D,F). Overall, the data suggest that upon WOX5 induction more cells in the upper columella undergo cell cycle progression and CSC-like cells divide more rapidly compared to control.

Interestingly, we observed the establishment of three domains in the enlarged columella of *35S:WOX5-GR* upon prolonged 48 h DEX induction: QC-like, CSC-like, and CSCD-like. At this stage, about five columella layers below the QC demonstrated the QC-like behavior, with the lack of EdU incorporation and mitoses (Figures 2C,F – red circle), and with highly dense nuclei similar to the QC in the wild type roots. The cells on their periphery continued to demonstrate CSC-like behavior with high DNA replication and mitotic activities (Figure 2F). The most distal CSCD-like layers generated during DEX exposure show weak traces of differentiation marking by minute starch granules and the absence of EdU-positive or mitotic nuclei.

Altogether these observations suggest that WOX5 overexpression does not lead to the dedifferentiation of DCCs, but regulates CSCs proliferation (both negatively and positively) and prevents CSCD differentiation.

### WOX5 Influences Auxin Response, Biosynthesis, and Transport in the Root Apical Meristem

If in wild type WOX5 acts as a local QC-specific regulator (Berckmans et al., 2020), but it might activate biosynthesis of the non-cell-autonomous signal auxin, we questioned, whether all mentioned above phenotypic changes in *35S:WOX5-GR* root tips are associated with the changes in auxin response, biosynthesis, and transport. Indeed, in agreement with the earlier findings (Tian et al., 2014b), we observed a gradual increase in the DR5 domain size and intensity in the root tips of *35S:WOX5-GR* plants carrying DR5:GFP reporter during DEX-incubation (Supplementary Figure 2).

**FIGURE 3:**
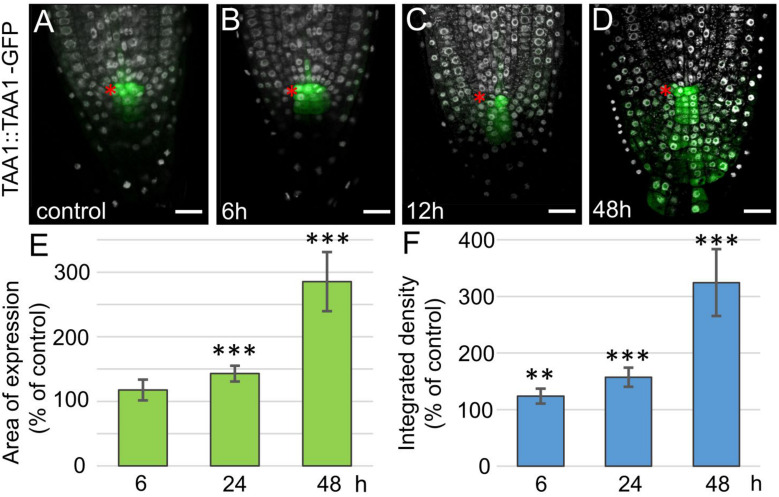
Increase in TAA1 expression in *35S:WOX5-GR* root tips during DEX exposure. **(A–D)** TAA1:TAA1-GFP expression at 0, 6, 12, and 48 h of DEX exposure (in green). DAPI is in white. The QC location is marked by the red asterisks. The bar scale – 20 μm. **(E,F)** Statistics on the intensity of TAA1-GFP fluorescence level in *35S:WOX5-GR* root tips after 6, 24, and 48 h of DEX exposure. The expression area **(E)** and the integrated density **(F)** of the fluorescent signal was measured using ImageJ (https://imagej.nih.gov/ij/docs/menus/analyze.html). The statistical significance of the differences relative to respected control, taken for each time point, was estimated using Welch’s *t*-test (**p < 0.01; ***p < 0.001; CI 95%, *N* = 20).

Tryptophan is converted to indole-3-acetic acid (IAA) in two-steps via indol-3-pyruvate (IPA), the first step is catalyzed by TAA1/TAR tryptophan aminotransferase enzymes, and the second step by flavin monooxygenases from YUCCA family (Mashiguchi et al., 2011; Won et al., 2011). The *WOX5* activatory role on *YUC1* has been proposed before (Tian et al., 2014b). Along with this, a non-significant increase in *TAA1* expression was shown by qPCR in a whole root. As TAA1, but not YUC1 expression domain greatly overlaps with WOX5 one, it was tempting to study if WOX5 regulates TAA1 expression. For this, we collected the root tips (3–4 mm in length) and performed qPCR analysis, on these samples we detected significant increase in TAA1 transcript after 24 h of DEX exposure (Supplementary Figure 3). To study the changes in the TAA1 expression domain, we introduced the TAA1:TAA1-GFP reporter into *35S:WOX5-GR* background (Figure 3A). As early as 6 h after DEX treatment and before any changes in the root tip anatomy become visible, TAA1-GFP fluorescence level starts increasing in the distal meristem (Figure 3B). TAA1-GFP expression domain begins expanding down to the root end after 12 h of DEX treatment, when CSCs start dividing (Figures 3C,E). TAA1-GFP expression domain further extends significantly up to 145 percent of the initial size after 24 h of DEX treatment. At 48 h of WOX5 induction, the TAA1 expression domain reaches the rootward end of the upper columella part (Figure 3D). Along with the expansion of the expression domain, an increase in TAA1 expression level is observed (Figure 3F).

**FIGURE 4:**
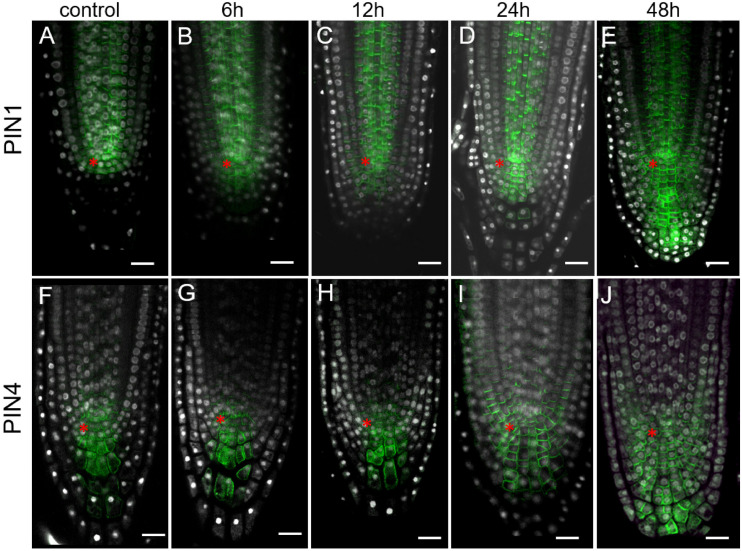
Expansion of PIN1 and PIN4 expression domains in *35S:WOX5-GR* root tips during DEX exposure. **(A–J)** Immunolocalization of PIN1 **(A–E)** and PIN4 **(F–J)** are in green at 0, 6, 12, 24, and 48 time points of DEX incubation. *N* = 10. DAPI is in white. The QC location is marked by the red asterisks. The bar scale – 20 μm.

As there is a positive correlation between the auxin level and expression of PIN auxin transporters (Vieten et al., 2005; Omelyanchuk et al., 2016; Brumos et al., 2018), but it was not detected in *35S:WOX5-GR* earlier (Tian et al., 2014b), we reanalyzed PINs expression pattern by immunolabeling. This analysis did not detect significant changes in PIN2 expression upon WOX5 induction confirming earlier findings (Tian et al., 2014b) (Supplementary Figure 4). However, we found significant differences in PIN1 and PIN4 expression patterns in *35S:WOX5-GR* as soon as 6 h after DEX exposure (Figures 4B,G). PIN1 and PIN4 expression domains extended downwards accompanying the outgrowth of the CSC-like cells in the upper columella. At 48 h of WOX5 induction, PIN1 and PIN4 proteins non-polarly localized throughout both QC-like, CSC-like and CSCD-like domains of columella (Figures 4E,J). The changes in PIN1 and PIN4 transcripts levels were also significant in the root tips after 6 and 24 h of DEX treatment as measured by qPCR (Supplementary Figure 3).

To sum up, we detected that both auxin synthesis, transport, and signaling enhanced upon WOX5 overexpression and these changes precede *35S:WOX5-GR* phenotype formation. Thus, we might hypothesize that it is not WOX5 itself, but accumulated in response to WOX5 auxin is the main factor provoking the columella phenotype formation. Next we tested this hypothesis with a mathematical modeling approach.

### Mathematical Modeling Demonstrated WOX5 Influence on Auxin Distribution in the Root Apical Meristem

**FIGURE 5:**
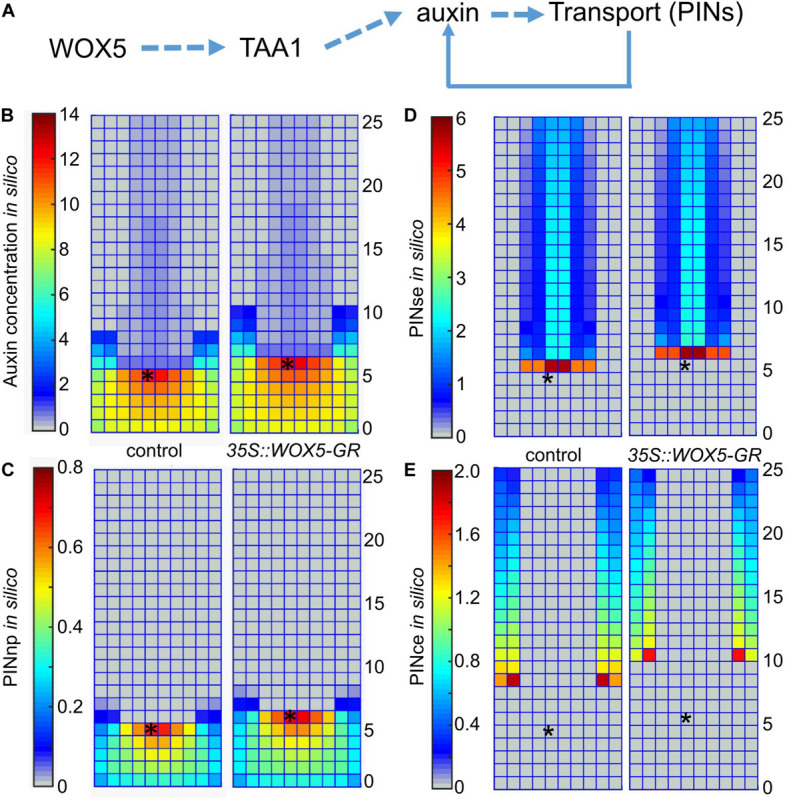
Simulation of auxin patterning in the root tip of control and *35S:WOX5-GR* plants after DEX exposure. **(A)** The hypothesis of WOX5 effect on auxin redistribution considered in the 2-dimensional mathematical model. **(B–E)** The steady state solutions for the 2D cell layout that simulates the longitudinal cut of the root tip in control and the *35S:WOX5-GR* plants after DEX exposure; **(B)** auxin distribution, **(C)** non-polarly localized auxin transporters in the root cap (PINnp), **(D)** rootward polarized auxin transporters in the stele and endodermis (PINse), **(E)** PINce transporter polarized rootward in the cortex and shootward in the epidermis. The QC location is marked by the black asterisks.

The experimental results (Figures 3, 4 and Supplementary Figures 2–4) indicate that WOX5 activates *de novo* auxin synthesis in the root tip, but also enhances its transportation and signaling. We used mathematical modeling to predict the changes in auxin distribution in the root tip considering multiple WOX5-mediated inputs. For that we applied *2*-dimensional model on auxin distribution in the root tip (Mironova et al., 2012; Hong et al., 2017), which considers positive and negative feedbacks between auxin and its three generalized transporters that mediate rootward (PINse in stele and endodermis), rootward/shootward (PINce in cortex and epidermis) and non-polar (PINnp in the root cap) auxin transportation in the root tip. In the model, we studied if an increase in the auxin biosynthesis rate induced by WOX5 overexpression may change the auxin transportation map (Figure 5A).

To simulate auxin patterning in wild type we used the parameter set from Hong et al. (2017) (Supplementary Table 3). Figures 5B,C (the left part) show the steady-state solution for auxin and non-polar auxin transporter (PINnp) patterns generated from the uniform initial data. To simulate auxin distribution in the *35S:WOX5-GR* root tip during DEX exposure, we started the calculation from the wild type steady-state solution (Figure 5B, left) with the auxin synthesis rate increased according to the fold-changes of TAA1 expression in *35S:WOX5-GR* compared to wild type (Figure 3F, Supplementary Table 3). This rapidly leads to auxin accumulation in the distal meristem (Figures 5B,C, right), followed by an increase in the expression of non-polar PINnp that nicely fit with the experimental observations (Figures 4, 5). The latter results in a slight increase in the auxin level in the proximal meristem, enhancement of the rootward auxin transport, and finally the shootward shift of the auxin maximum location. The shift in auxin maximum location (Figure 5B) suggests an increase in the number of columella layers.

Thus, our simulation demonstrates that WOX5-mediated increase in TAA1 expression might be sufficient to reproduce all the differences in PINs and auxin patterning between the control and DEX-induced *35S:WOX5-GR* root tips.

### Mathematical Modeling Showed That WOX5 Can Regulate the Distal Meristem Functioning by Adjusting Local Auxin Biosynthesis

**FIGURE 6:**
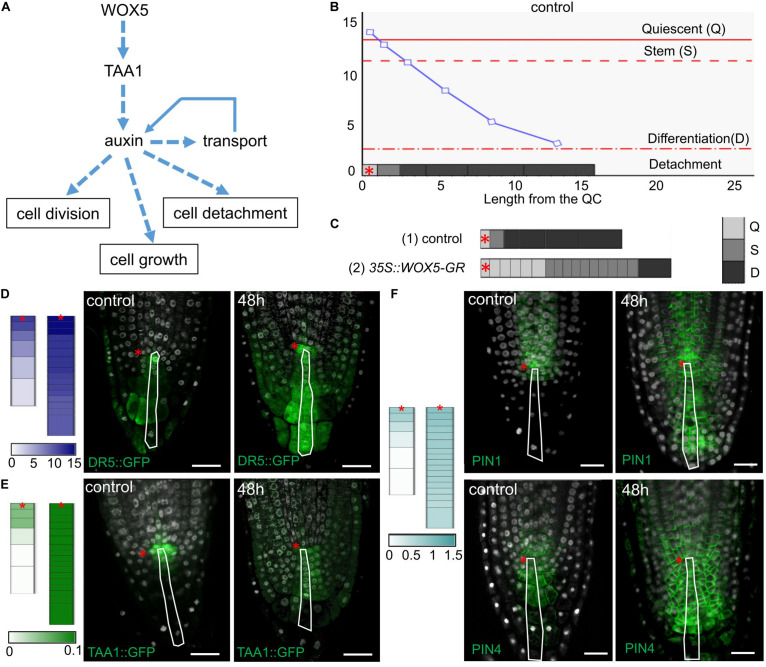
Simulation of cell dynamics in the distal meristem of the *35S:WOX5-GR* root tips. **(A)** The cell dynamics regulations considered in the 1-dimensional model. **(B)** Simulated auxin concentration in a cell determines its differentiation state. The *X*-axis denotes the columella length and demonstrates its structure (the QC length in wild type is taken as 1). The gray rectangles on the *x-*axis display the columella layout with the cells of three following states: *Quiescent (Q)* – cells can’t grow and divide, *Stem (S)* – cells can grow and divide, and *Differentiation (D)* – cells can only grow. The cell states are color-coded in gray scale. The fourth state – detached cells are outside the layout. *Y*-axis indicates calculated auxin concentration in concentration units (*cu*). The blue curve represents changes in auxin concentration in the layout. Red lines depict the thresholds for state changes depending on auxin concentration. The QC location is marked by the red asterisks. **(C)** Calculation results for the cell ensembles in control (quasi-balance mode, Supplementary Video 1) and DEX-induced *35S:WOX5-GR* (broken-balance mode, Supplementary Video 3) root tips at 1,500 calculation steps. The colored rectangles display the cell ensemble generated until this calculation step. Gray color intensity reflects the cell states. The QC location is marked by the red asterisks. **(D–F)** Comparison of calculation results for control (Supplementary Video 1) and DEX-induced *35S:WOX5-GR* (Supplementary Video 3) root tips taken at 1,600 calculation steps with the experimental data. Color intensity of the colored rectangles reflects *in silico* concentration units for TAA1 (green), PINnp (light blue), auxin (navy blue). **(D)** Auxin concentration levels versus experimental data on auxin response levels (from Supplementary Figure 2). **(E)** TAA1 expression (Figure 3). **(F)** PINnp expression levels versus immuno-labeling of PIN1 and PIN4 proteins (Figure 4).

Local auxin biosynthesis plays a key role in the root meristem functions (Brumos et al., 2018). To test how an increase in the local auxin biosynthesis rate would change the cell dynamics in the columella we created a *1*-dimensional model simulating cell dynamics in the QC, CSC, and columella cells located along the central root axis (Figure 6A and Supplementary Material). The model reproduces the cell dynamics rules proposed for the columella in Dubreuil et al. (2018), where the cells grow continuously between the discrete events of cell division and detachment. In our model these events depend on the internal auxin concentration (*a*_i_) with the thresholds for cell division (*s*_CSC_) and cell detachment (*s*_D_). To take into account the distinct behavior of the QC, we added a rule for cessation of cell growth depending on the auxin level with the threshold *s*_QC_. Thus, the model is capable to generate four auxin-dependent cell states: the *Quiescent state (Q)*, when *a_i_* ≥ *s*_QC_; the *Stem state (S)*, when *s_CSC_* ≤ *a_i_* < s*_QC_*; the *Differentiation state (D)*, when *s_D_* ≤ *a_i_* < s*_CSC_*; and the *Detachment state* for removing the cell from the ensemble if *a_i_* < *s*_D_ (Figure 6B). Besides the auxin flow from the shoot we added into the model the TAA1 protein, which provided for the WOX5-mediated local source of auxin.

We adjusted the model parameters so that the model reproduced well the cell dynamics in the distal meristem of wild type roots (Figure 6B, Supplementary Tables 4, 5, and Supplementary Video 1). Namely, both CSC division and detachment of the last DCC reiterated, so that a “quasi-balanced” dynamics in columella occurred despite intense cell growth, division and detachment processes. During calculation, we always observed one non-growing and non-dividing cell corresponding to the QC, one growing and capable to divide cell corresponding to the CSC, and four (very rarely five) non-dividing, but growing cells, corresponding to the DCCs. Herewith, the auxin distribution specific for wild type was retained, and TAA1 expression was observed in the QC and one or two neighboring cells.

To simulate columella cell dynamics in *35S:WOX5-GR* root tips upon DEX treatment, we varied the TAA1 biosynthesis rate within the confidence interval of TAA1 expression changes after 24 h of DEX exposure (Figure 3F, Supplementary Table 4). Despite the fact that we changed the value of only one parameter, we got a great alteration in the columella cells dynamics. Upon even slight changes of TAA1 biosynthesis rate, the distal meristem enlarged, and got a new “quasi-balanced” dynamics with several additional CSC and QC layers (Supplementary Video 2). When we increased the auxin biosynthesis rate two times, the balance between CSC divisions and the last DCC detachment became broken; abundant cell divisions occur (Supplementary Video 3). In this “broken-balance” mode the pool of the cells capable of dividing expanded in time, because CSCDs did not lose this capability (*a_i_* ≥ *s*_CSC_). Furthermore, we observed an increased number of cells in the Quiescent state (*a_i_* > *s*_QC_), in agreement with the experimental observations (Figure 2F, red circle) that not all the cells within the enlarged distal meristem had the same CSC identity (Figure 6C, Supplementary Video 3). Moreover, auxin distribution and expression pattern of PINnp in the “broken-balance” mode of the model qualitatively match the expression pattern of DR5, PIN1, PIN4 reporter lines in columella for *35S:WOX5-GR* observed experimentally (Figures 6D–F).

Modeling of the cell dynamics in the distal meristem confirms that WOX5-mediated increase in TAA1-dependent auxin biosynthesis is sufficient to reproduce the *35S:WOX5-GR* columella phenotype.

### Inhibition of TAA1-Mediated Auxin Synthesis Opens the Meristem in *wox5-1* Mutant and Partially Rescues the *35S:WOX5-GR* Columella Phenotype

One-dimensional model gave an interesting output when decreasing TAA1 synthesis rate: decrease by 50% led to a new “quasi-balanced” dynamics without any cells in the *Quiescent state*, but with one or two cells in the *Stem state* (Supplementary Video 4). We speculated that this behavior happens *in vivo* in the *wox5* knockout line (Supplementary Figure 5). Indeed, QC divisions were described for *wox5-1* mutant (Forzani et al., 2014). Opening of the root meristem in *wox5-1* (Supplementary Figure 5F), however, does not have a major influence on the root growth, as *wox5-1* primary roots are only slightly shorter compared to wild-type (Supplementary Figure 6). In the crossings of *wox5-1* with TAA1:TAA1-GFP and DR5:GFP reporter lines, we detected the DR5 signal and TAA1 expression lower than in wild type, that confirmed the model prediction (Supplementary Figures 5G,H). We also questioned if an inhibition of TAA1-dependent auxin synthesis might rescue columella phenotype of *35S:WOX5-GR*, at least partially. In the *1*-dimensional model a 50% inhibition of TAA1-mediated auxin synthesis does recover the columella phenotype to wild type (Supplementary Video 5).

**FIGURE 7:**
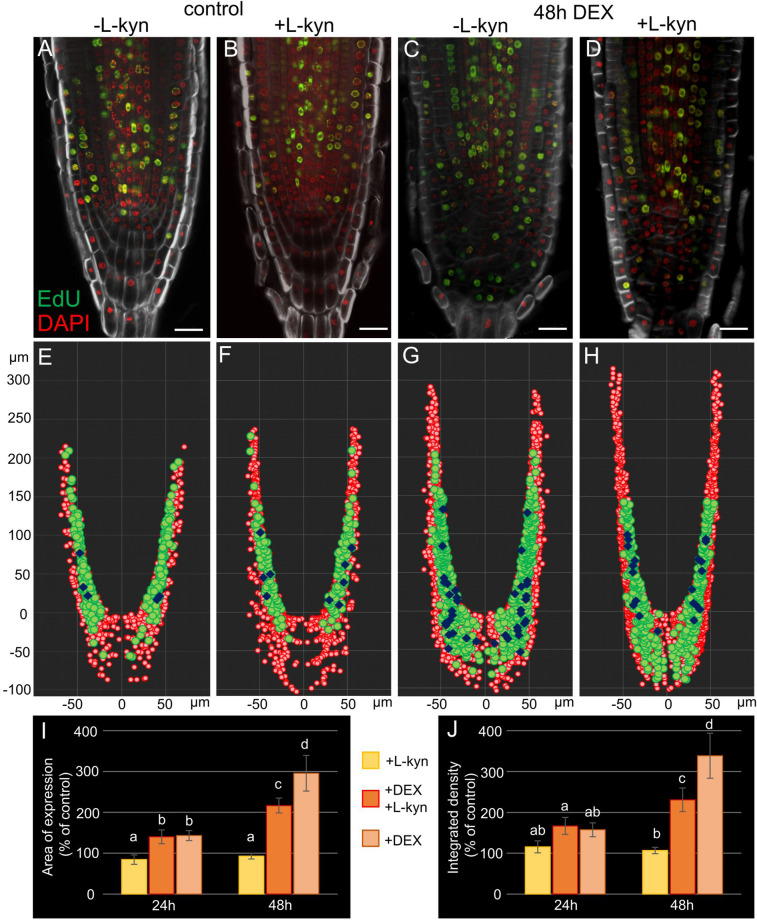
Auxin synthesis inhibitor L-kynurenine partially restores the wild type root tip phenotype in DEX-induced *35S:WOX5-GR* plants. **(A–D)** EdU-labeling of *35S:WOX5-GR* root tips after 48 h treatment with mock **(A)**, L-kynurenine **(B)**, DEX **(C)**, and DEX with L-kynurenine **(D)**. EdU is in green counterstained with DAPI in red and BR28 in white. The bar scale – 20 μm. **(E–H)** The nuclei distribution maps for the representative root tips *of 35S:WOX5-GR* **(A–D)** quantitatively analyzed in 3D with iRoCs Toolbox (Schmidt et al., 2014). The nuclei projections to the longitudinal cut are depicted as red circles, EdU-labeled nuclei as green circles, mitotic ones as blue rhombs. **(I,J)** Quantitative analysis of the expression area **(I)** and integrated fluorescence density **(J)** in *35S:WOX5-GR* TAA1:TAA1-GFP root tips. The TAA1-GFP fluorescence changes measured upon L-kynurenine exposure (+ L-kyn), simultaneous action of L-kynurenine and dexamethasone (+ DEX + L-kyn), and dexamethasone exposure (+ DEX) after 24 and 48 h relative to the mock treatments. The statistical significance was estimated by one-way ANOVA with Tukey *post hoc* test (CI 95%, *N* = 20): letters “a,” “b,” “c,” and “d” denote significantly different groups of samples.

To verify this experimentally we added L-kynurenine, a competitive inhibitor of TAA1/TAR-dependent auxin biosynthesis (He et al., 2011), into the media containing DEX (see “Materials and Methods”). qPCR analysis of the root tips exposed to combined DEX/L-kynurenine treatment for 24 h showed dampened response relative to DEX treatment for TAA1, PIN1, and PIN4 (Supplementary Figure 3). Quantitative analysis of TAA1-GFP fluorescence showed a smaller induction of TAA1 after 48 h of combined DEX/L-kynurenine action relative to DEX only (Figures 7I,J). The difference was even higher if we consider that there are two types of *35S:WOX5-GR* TAA1:TAA1-GFP plants after DEX/L-kynurenine treatment: the major part of plants (66%, *N* = 30) showed normal or slightly wider TAA1 expression domain, while the rest plants showed dramatic increase in TAA1 expression domain without signs of recovery (Supplementary Figure 7).

Partial rescue columella phenotype of *35S:WOX5-GR* plants under DEX/L-kynurenine became 48 h after treatment (Figure 7, Supplementary Figure 7). Namely, these roots develop less root cap layers, CSCDs become able to differentiate. Quantitative analysis of distribution of mitotic and EdU-positive cells with iRoCS Toolbox (Schmidt et al., 2014) (Figures 7E–H) showed that L-kynurenine dampened extra mitotic activity in the distal meristem, but enhanced it back to normal in the proximal one (Table 1).

**TABLE 1:** Quantitative analysis of mitosis distribution in the *35S:WOX5-GR* root tips before and after 48 h of DEX incubation, with and without auxin synthesis inhibitor L-kynurenine.

	**Mitosis number**	**− L-kynurenine (48 h)**	**+ L-kynurenine (48 h)**
Control	Distal meristem/root cap	5	9
	Proximal meristem	132	105
*35S:*	Distal meristem/root cap	38	20
*WOX5-GR*	Proximal meristem	49	89

## Discussion

WOX5 belongs to the plant-specific subclade of the homeobox transcription factor superfamily (van der Graaff et al., 2009). Highly conserved among the angiosperms *WUS* and *WOX5* genes have similar, interchangeable roles in the maintenance of the stem cell niche in the shoot and root meristems, correspondingly (Sarkar et al., 2007; van der Graaff et al., 2009). Despite the mechanisms of WUS/WOX5 expression domains positioning are well studied (Jönsson et al., 2005; Tian et al., 2014b; Pi et al., 2015; Zhang et al., 2015), the information about WUS/WOX5 downstream targets is poor. In shoot apical meristem, WUS rheostatically controls auxin response and signaling to maintain stem cells, and low auxin level is required for stem cell self-renewing whereas local auxin accumulation triggers differentiation (Vernoux et al., 2000; Ma et al., 2019). It is different in the root apical meristem, auxin maximum is required for stem cell maintenance and for differentiation of some stem cell daughters, for example, CSCDs (Sabatini et al., 1999; Blilou et al., 2005). Also, auxin acts both up- and downstream of WOX5 and auxin signaling is controlled by WOX5 via its influence on auxin biosynthesis (Ding and Friml, 2010; Tian et al., 2014b). First it was found that YUC1 is one of WOX5 downstream targets because YUC1 was activated in *35S:WOX5-GR* seedlings by DEX treatment (Tian et al., 2014b). Here we show that WOX5 influence on auxin biosynthesis is enhanced by affecting also TAA1 expression. Furthermore, by mathematical modeling we demonstrated that WOX5-mediated increase in TAA1 expression is sufficient to reproduce differences in PINs expression, auxin patterning and columella structure between the wild type and *35S:WOX5-GR* induced DEX roots.

Mathematical modeling has been shown as an efficient method to study auxin distribution in plants (reviewed in Morales-Tapia and Cruz-Ramírez, 2016). And it has already been applied to study WOX5 functioning (Tian et al., 2014b). The computer simulation demonstrated that the WOX5-IAA17-ARF10/16 regulatory circuit (linking IAA17-mediated *WOX5* activation via suppression of repressors, WOX5-modulated auxin synthesis with auxin-regulated IAA17 expression) is required for both auxin maximum and distal stem cell niche function in the root tip. The model used in Tian et al. (2014b) neither considers auxin-regulated PINs expression nor auxin-regulated cell dynamics. Our results complement the findings shown by Tian et al. (2014b) with the data on self-adjustment of auxin transportation map and different modes of cell dynamics depending on the efficiency of WOX5-mediated auxin synthesis rates. Also in good agreement with (Brumos et al., 2018), it was shown that local auxin biosynthesis in the QC is crucial for the stem cell niche maintenance.

We also showed that depending on the efficiency of TAA1 activation there might be several modes of root meristem functioning. Relatively low TAA1 activation leads to formation of an “open meristem” without the QC as in *wox5-1* knockout mutant (Supplementary Figure 3, Supplementary Video 4). Moderate TAA1 activation leads to formation and maintenance of the distal meristems with one or several CSC and QC layers (Supplementary Videos 1, 2). This result predicts that higher auxin synthesis rates in the root meristem would lead to formation of thicker meristems, like in maize or rice, the species with less studied mechanisms of root meristem maintenance. Finally, the model predicts that high auxin synthesis rate would break the stem cell niche self-sustaining balance leading to the outgrowth with many QC-like and CSC-like cells, like we observe in *35S:WOX5-GR* (Figure 6C(2), Supplementary Video 3) or like other authors observed upon callus induction or organ regeneration (reviewed in Ikeuchi et al., 2019; Sugimoto et al., 2019). As the efficiency of regeneration in plants depends very much on the genetic background, this might mean the plants with a poor ability to regenerate might not induce the endogenous auxin synthesis rates enough.

All these data make TAA1 a prospective candidate for the WOX5 key target, either direct or indirect. The fact that WOX5 upregulates the enzymes for both steps of IAA synthesis explain why it is so important for development of primary and lateral roots (Tian et al., 2014a), as well as for plant regeneration processes (reviewed in Ikeuchi et al., 2019; Sugimoto et al., 2019).

## Conclusion

The stem cell niche in the root apical meristem due to its close to geometrically regular structure is one of the most suitable objects for studying the mechanisms of keeping the balance between stem cell self-renewal and differentiation. Here we studied in detail the interaction between WOX5, the key transcription factor attributing to root tip stem cell niche organizing center and auxin, the phytohormone mostly involved in plant development. We demonstrated both *in vivo* and *in silico* that WOX5 activation of the TAA1 gene encoding enzyme for the first stage of auxin biosynthesis is sufficient to provide increase in local auxin biosynthesis followed by increase in auxin level, auxin redistribution and changes in columella cell self-renewing and differentiation. By this we provide evidence that WOX5-TAA1-auxin circuit is one of the key parts of the complex gene network guiding maintenance of CSCs and columella development.

## Data Availability Statement

The original contributions presented in the study are included in the article/Supplementary Material, further inquiries can be directed to the corresponding author/s.

## Author Contributions

TP, KP, VVM, and VVL conceived and designed the experiments. TP and DDN performed the experiments. VVL, VVM, and NAO analyzed the data. MSS developed mathematical models and performed simulations. All authors contributed in writing the manuscript.

## Conflict of Interest

The authors declare that the research was conducted in the absence of any commercial or financial relationships that could be construed as a potential conflict of interest.

## Footnotes

1 www.thermofisher.com/

2 http://imagej.nih.gov/ij

## Supplementary Material

Supplementary Figure 1 Interface of iRoCS Toolbox. The root tip scanned in 3D and annotated with iRoCS Toolbox. Three projections with overlaid color-coded annotations are shown in the iRoCS interface. Colored circles denote nuclei of different tissues. Color map is superimposed in the center of the figure.

Supplementary Figure 2 Increase of the DR5 signal in *35S:WOX5-GR* root tips during DEX exposure at 0, 6, and 48 h time points. Auxin response was visualized by DR5:GFP (in green). DAPI displays nuclei in white. The bar scale – 20 μm.

Supplementary Figure 3 TAA1, PIN1, and PIN4 transcription is induced upon DEX treatment in *35S:WOX5-GR* root tips. qRT-PCR analysis of TAA1 **(A)**, PIN1 **(B)**, PIN4 **(C)** expression in roots after 6 h, 24 h DEX, and after 24 h DEX + L-kynurenine exposure. Data represent fold changes in transcript levels compared those in mock-treated plants. Error bars show SD. Three biologically independent samples were analyzed per treatment and per time point. Statistical significance was done by Welch’s *t*-test (^∗^*p* < 0.05; ^∗∗^*p* < 0.01; ^∗∗∗^*p* < 0.001).

Supplementary Figure 4 PIN2 expression domain did not change significantly in *35S:WOX5-GR* root tips after 48 h of DEX exposure, except being additionally expressed in the extra layers of the lateral root cap. Immunolocalization for PIN2 at 0 and 48 h time points of DEX exposure. DAPI is in white. The QC location is marked by the red asterisks. Blue arrows mark lateral root cap layers with PIN2 expression under the QC position. The bar scale – 20 μm.

Supplementary Figure 5 Knockout mutant *wox5-1* compared to wild type. **(A)** Anatomy of wild type root tip. **(B)** DR5:GFP expression in wild type root tip. **(C)** TAA1:TAA1-GFP expression in wild type root tip. **(D)** Immunolocalization of PIN1 in wild type root tip. **(E)** Immunolocalization of PIN4 in wild type root tip. **(F)** Anatomy of *wox5-1* root tip. **(G)** DR5:GFP expression in *wox5-1* root tip. **(H)** TAA1:TAA1-GFP expression in *wox5-1* root tip. **(I)** Immunolocalization of PIN1 in *wox5-1* root tip. **(J)** Immunolocalization of PIN4 in *wox5-1* root tip. **(K)** Calculation results for the cell ensembles in wild type and *wox5-1* root tips at 1,500 calculation steps. The gray rectangles display the cell states [*Quiescent (Q)*, *Stem (S)*, and *Differentiation (D)*]. The QC location is marked by the red asterisks.

Supplementary Figure 6 Shortening of the *wox5-1* primary root compared to the wild type. The statistical significance of the differences was estimated using Welch’s *t*-test (^∗^*p* < 0.05; ^∗∗^*p* < 0.01; ^∗∗∗^*p* < 0.001).

Supplementary Figure 7 L-Kynurenine partially rescue a portion of DEX-treated 35S:WOX5-GR TAA1:TAA1-GFP plants. **(A)** Mock-treated plants. **(B)** TAA1-GFP signal upon 48 h DEX treatment. **(C)** TAA1-GFP is partially rescued by L-kynurenine plants (66%, *N* = 20). **(D)** No rescue occurs in 33% of plants treated by L-kynurenine. The QC location is marked by the red asterisks. The bar scale – 20 μm.

Supplementary Dataset 1 Exported data from iRoCS Toolbox of three roots for Figure 2.

Supplementary Table 1 Quantitative analysis of mitotic and S-phase nuclei distributions in 35S:WOX5-GR root tips at 0, 12, and 48 h of DEX incubation. Analysis performed on the root tip models created in iRoCs Toolbox (Schmidt et al., 2014) using 3D confocal images labeled by EdU/DAPI.

Supplementary Table 2 The primers used for qRT-PCR.

Supplementary Table 3 Parameter settings used in the 2D model.

Supplementary Table 4 Cell sizes for the QC and the columella cells used as initial data.

Supplementary Table 5 Parameter settings used in the 1D model.

## References

[B1] Berckmans B. Kirschner G. Gerlitz N. Stadler R. Simon R. (2020). CLE40 signaling regulates root stem cell fate. *Plant Physiol.* 182 1776–1792. 10.1104/pp.19.00914 31806736 PMC7140941

[B2] Blilou I. Xu J. Wildwater M. Willemsen V. Paponov I. Friml J. (2005). The PIN auxin efflux facilitator network controls growth and patterning in Arabidopsis roots. *Nature* 433 39–44. 10.1038/nature03184 15635403

[B3] Brumos J. Robles L. M. Yun J. Vu T. C. Jackson S. Alonso J. M. (2018). Local auxin biosynthesis is a key regulator of plant development. *Dev. Cell* 47 306–318. 10.1016/j.devcel.2018.09.022 30415657

[B4] Ding Z. Friml J. (2010). Auxin regulates distal stem cell differentiation in Arabidopsis roots. *Proc. Natl. Acad. Sci. U.S.A.* 107 12046–12051. 10.1073/pnas.1000672107 20543136 PMC2900669

[B5] Dubreuil C. Jin X. Grönlund A. Fischer U. (2018). A local auxin gradient regulates root cap self-renewal and size homeostasis. *Curr. Biol.* 28 2581–2587. 10.1016/j.cub.2018.05.090 30078563

[B6] Forzani C. Aichinger E. Sornay E. Willemsen V. Laux T. Dewitte W. (2014). WOX5 suppresses CYCLIN D activity to establish quiescence at the center of the root stem cell niche. *Curr. Biol.* 24 1939–1944. 10.1016/j.cub.2014.07.019 25127220 PMC4148176

[B7] He W. Brumos J. Li H. Ji Y. Ke M. Gong X. (2011). A small-molecule screen identifies L-kynurenine as a competitive inhibitor of TAA1/TAR activity in ethylene-directed auxin biosynthesis and root growth in Arabidopsis. *Plant Cell* 23 3944–3960. 10.1105/tpc.111.089029 22108404 PMC3246337

[B8] Hong J. H. Savina M. Du J. Devendran A. Ramakanth K. K. Tian X. (2017). A sacrifice-for-survival mechanism protects root stem cell niche from chilling stress. *Cell* 170 102–113. 10.1016/j.cell.2017.06.002 28648662

[B9] Ikeuchi M. Favero D. S. Sakamoto Y. Iwase A. Coleman D. Rymen B. (2019). Molecular mechanisms of plant regeneration. *Annu. Rev. Plant Biol.* 70 377–406. 10.1146/annurev-arplant-050718-100434 30786238

[B10] Jönsson H. Heisler M. Reddy G. V. Agrawal V. Gor V. Shapiro B. E. (2005). Modeling the organization of the WUSCHEL expression domain in the shoot apical meristem. *Bioinformatics* 21(Suppl._1), i232–i240. 10.1093/bioinformatics/bti1036 15961462

[B11] Lavrekha V. V. Pasternak T. Palme K. Mironova V. V. (2020). 3D analysis of mitosis distribution pattern in the plant root tip with iRoCS toolbox. in *Plant Stem Cells. Methods in Molecular Biology*. Vol. 2094, eds Naseem M. Dandekar T. (New York, NY: Humana), 119–125. 10.1007/978-1-0716-0183-9_13 31797297

[B12] Ma Y. Miotk A. Šutiković Z. Ermakova O. Wenzl C. Medzihradszky A. (2019). WUSCHEL acts as an auxin response rheostat to maintain apical stem cells in Arabidopsis. *Nat. Commun.* 10:5093. 10.1038/s41467-019-13074-9 31704928 PMC6841675

[B13] Mashiguchi K. Tanaka K. Sakai T. Sugawara S. Kawaide H. Natsume M. (2011). The main auxin biosynthesis pathway in Arabidopsis. *Proc. Natl. Acad. Sci. U.S.A.* 108 18512–18517. 10.1073/pnas.1108434108 22025724 PMC3215075

[B14] Mironova V. V. Omelyanchuk N. A. Novoselova E. S. Doroshkov A. V. Kazantsev F. V. Kochetov A. V. (2012). Combined in silico/in vivo analysis of mechanisms providing for root apical meristem self-organization and maintenance. *Ann. Bot.* 110 349–360. 10.1093/aob/mcs069 22510326 PMC3394645

[B15] Morales-Tapia A. Cruz-Ramírez A. (2016). Computational modeling of auxin: a foundation for plant engineering. *Front. Plant Sci.* 20:1881. 10.3389/fpls.2016.01881 28066453 PMC5168462

[B16] Omelyanchuk N. A. Kovrizhnykh V. V. Oshchepkova E. A. Pasternak T. Palme K. Mironova V. V. (2016). A detailed expression map of the PIN1 auxin transporter in Arabidopsis thaliana root. *BMC Plant Biol.* 16:5. 10.1186/s12870-015-0685-0 26821586 PMC4895256

[B17] Pasternak T. Tietz O. Rapp K. Begheldo M. Nitschke R. Ruperti B. (2015). Protocol: an improved and universal procedure for whole-mount immunolocalization in plants. *Plant Methods* 11:50. 10.1186/s13007-015-0094-2 26516341 PMC4625903

[B18] Pi L. Aichinger E. van der Graaff E. Llavata-Peris C. I. Weijers D. Hennig L. (2015). Organizer-derived WOX5 signal maintains root columella stem cells through chromatin-mediated repression of CDF4 expression. *Dev. Cell* 33 576–588. 10.1016/j.devcel.2015.04.024 26028217

[B19] Richards S. Wink R. H. Simon R. (2015). Mathematical modelling of WOX5-and CLE40-mediated columella stem cell homeostasis in Arabidopsis. *J. Exp. Bot.* 66 5375–5384. 10.1093/jxb/erv257 26019259 PMC4526915

[B20] Sabatini S. Beis D. Wolkenfelt H. Murfett J. Guilfoyle T. Malamy J. (1999). An auxin-dependent distal organizer of pattern and polarity in the Arabidopsis root. *Cell* 99 463–472. 10.1016/s0092-8674(00)81535-4 10589675

[B21] Sarkar A. K. Luijten M. Miyashima S. Lenhard M. Hashimoto T. Nakajima K. (2007). Conserved factors regulate signalling in Arabidopsis thaliana shoot and root stem cell organizers. *Nature* 446 811–814. 10.1038/nature05703 17429400

[B22] Schmidt T. Pasternak T. Liu K. Blein T. Aubry-Hivet D. Dovzhenko A. (2014). The iRoCS T oolbox–3 D analysis of the plant root apical meristem at cellular resolution. *Plant J.* 77 806–814. 10.1111/tpj.12429 24417645

[B23] Stahl Y. Grabowski S. Bleckmann A. Kühnemuth R. Weidtkamp-Peters S. Pinto K. G. (2013). Moderation of Arabidopsis root stemness by CLAVATA1 and Arabidopsis CRINKLY4 receptor kinase complexes. *Curr. Biol.* 23 362–371. 10.1016/j.cub.2013.01.045 23394827

[B24] Stahl Y. Simon R. (2009). Is the Arabidopsis root niche protected by sequestration of the CLE40 signal by its putative receptor ACR4? *Plant Signal Behav.* 4 634–635. 10.4161/psb.4.7.8970 19820344 PMC2710560

[B25] Sugimoto K. Temman H. Kadokura S. Matsunaga S. (2019). To regenerate or not to regenerate: factors that drive plant regeneration. *Curr. Opin. Plant Biol.* 47 138–150. 10.1016/j.pbi.2018.12.002 30703741

[B26] Tian H. Jia Y. Niu T. Yu Q. Ding Z. (2014a). The key players of the primary root growth and development also function in lateral roots in Arabidopsis. *Plant Cell Rep.* 33 745–753. 10.1007/s00299-014-1575-x 24504658

[B27] Tian H. Wabnik K. Niu T. Li H. Yu Q. Pollmann S. (2014b). WOX5–IAA17 feedback circuit-mediated cellular auxin response is crucial for the patterning of root stem cell niches in Arabidopsis. *Mol. Plant* 7 277–289. 10.1093/mp/sst118 23939433

[B28] van den Berg C. Willemsen V. Hendriks G. Weisbeek P. Scheres B. (1997). Short-range control of cell differentiation in the Arabidopsis root meristem. *Nature* 390 287–289. 10.1038/36856 9384380

[B29] van der Graaff E. Laux T. Rensing S. A. (2009). The WUS homeobox-containing (WOX) protein family. *Genome Biol.* 10:248. 10.1186/gb-2009-10-12-248 20067590 PMC2812940

[B30] Vernoux T. Kronenberger J. Grandjean O. Laufs P. Traas J. (2000). PIN-FORMED 1 regulates cell fate at the periphery of the shoot apical meristem. *Development* 127 5157–5165. 11060241 10.1242/dev.127.23.5157

[B31] Vieten A. Vanneste S. Wiśniewska J. Benková E. Benjamins R. Beeckman T. (2005). Functional redundancy of PIN proteins is accompanied by auxin-dependent cross-regulation of PIN expression. *Development* 132, 4521–4531. 10.1242/dev.02027 16192309

[B32] Wang J. W. Wang L. J. Mao Y. B. Cai W. J. Xue H. W. Chen X. Y. (2005). Control of root cap formation by microRNA-targeted auxin response factors in Arabidopsis. *Plant Cell* 17 2204–2216. 10.1105/tpc.105.033076 16006581 PMC1182483

[B33] Won C. Shen X. Mashiguchi K. Zheng Z. Dai X. Cheng Y. (2011). Conversion of tryptophan to indole-3-acetic acid by tryptophan aminotransferases of Arabidopsis and YUCCAs in Arabidopsis. *Proc. Natl. Acad. Sci. U.S.A* 108 18518–18523. 10.1073/pnas.1108436108 22025721 PMC3215067

[B34] Zhang Y. Jiao Y. Liu Z. Zhu Y. X. (2015). ROW1 maintains quiescent centre identity by confining WOX5 expression to specific cells. *Nat. commun.* 6:6003. 10.1038/ncomms7003 25631790 PMC4316744

