# Supplementary figures and images for: Cell Dynamics in WOX5-Overexpressing Root Tips: The Impact of Local Auxin Biosynthesis
Source: Front Plant Sci. 2020 Oct 22;11:560169. doi: 10.3389/fpls.2020.560169 (PMC7642516; doi:10.3389/fpls.2020.560169)

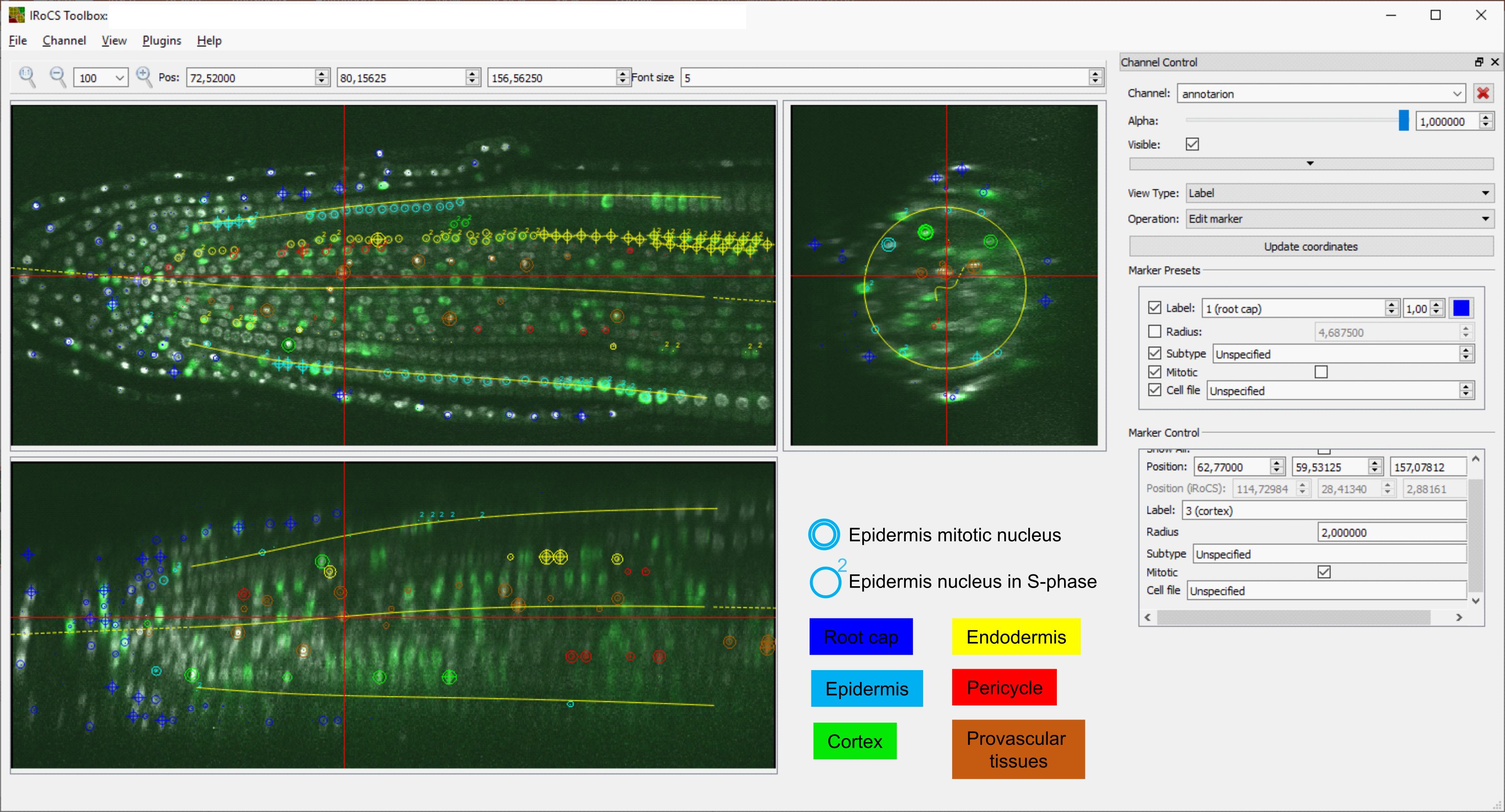

Supplement: Supplementary Figure 1 — Interface of iRoCS Toolbox. The root tip scanned in 3D and annotated with iRoCS Toolbox. Three projections with overlaid color-coded annotations are shown in the iRoCS interface. Colored circles denote nuclei of different tissues. Color map is superimposed in the center of the figure. [file Image_1.JPEG]

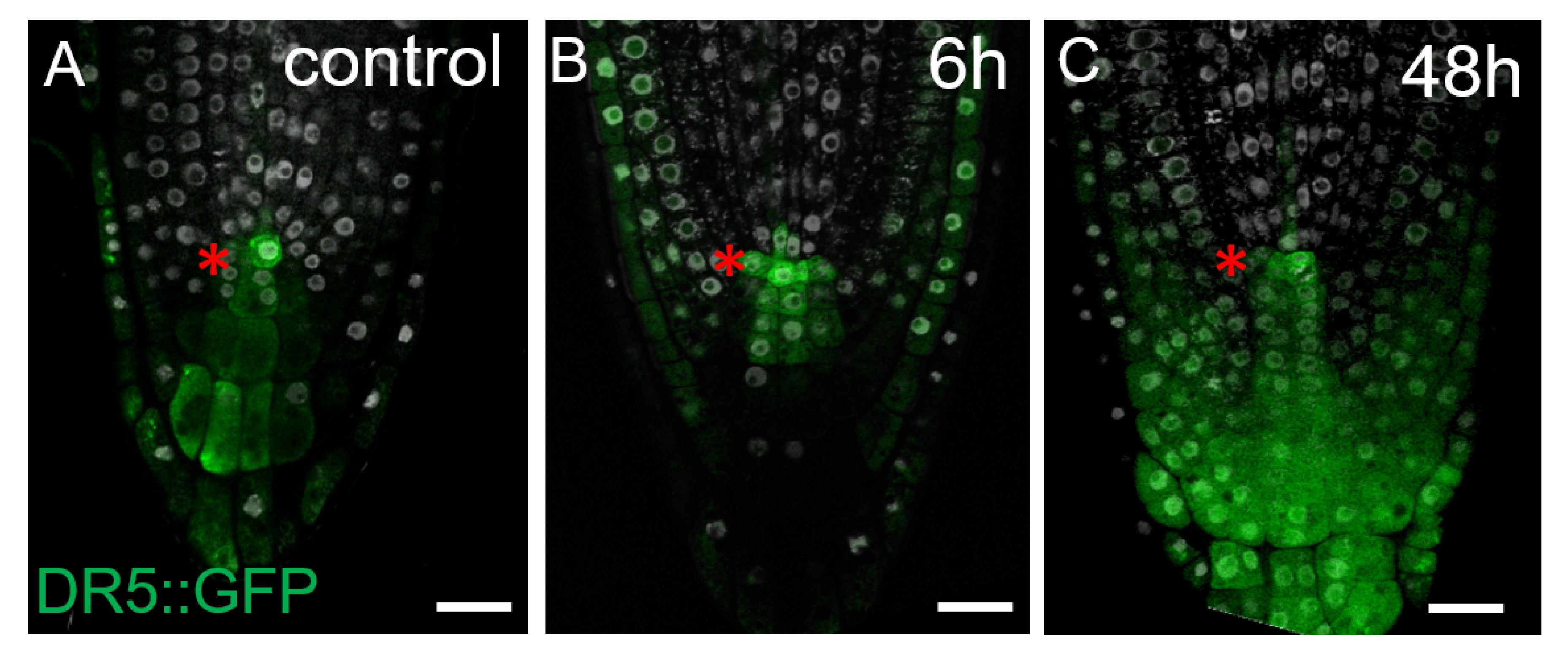

Supplement: Supplementary Figure 2 — Increase of the DR5 signal in 35S:WOX5-GR root tips during DEX exposure at 0, 6, and 48 h time points. Auxin response was visualized by DR5:GFP (in green). DAPI displays nuclei in white. The bar scale – 20 μm. [file Image_2.JPEG]

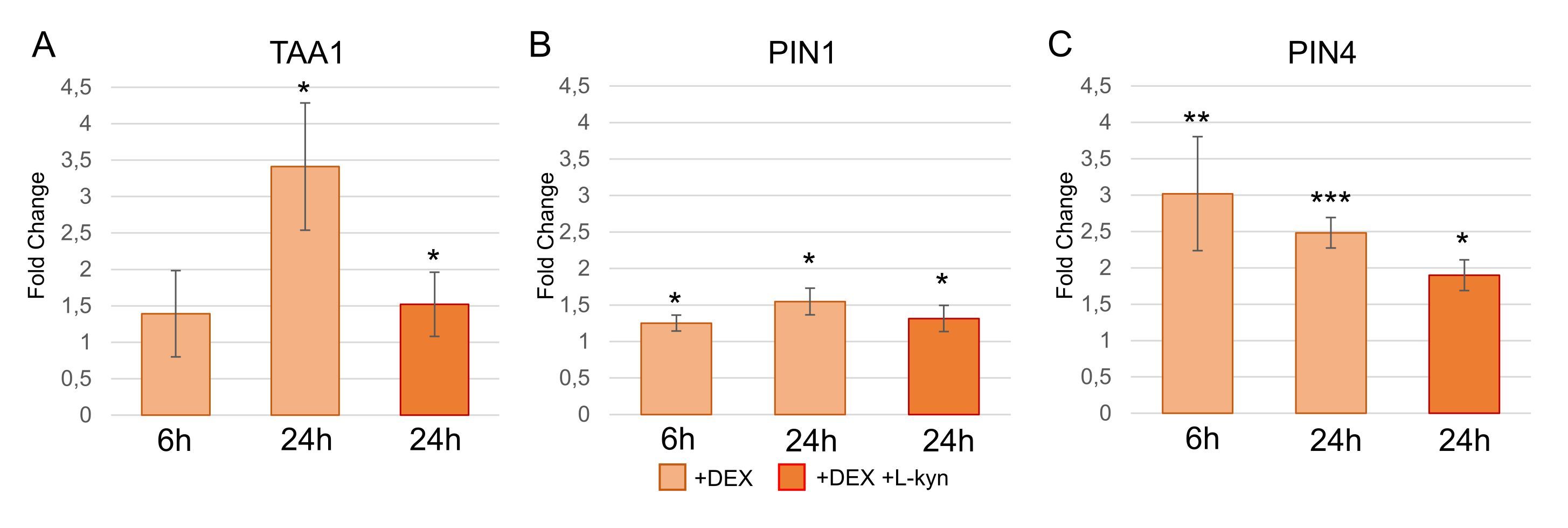

Supplement: Supplementary Figure 3 — TAA1, PIN1, and PIN4 transcription is induced upon DEX treatment in 35S:WOX5-GR root tips. qRT-PCR analysis of TAA1 (A), PIN1 (B), PIN4 (C) expression in roots after 6 h, 24 h DEX, and after 24 h DEX + L-kynurenine exposure. Data represent fold changes in transcript levels compared those in mock-treated plants. Error bars show SD. Three biologically independent samples were analyzed per treatment and per time point. Statistical significance was done by Welch’s t-test (∗p < 0.05; ∗∗p < 0.01; ∗∗∗p < 0.001). [file Image_3.JPEG]

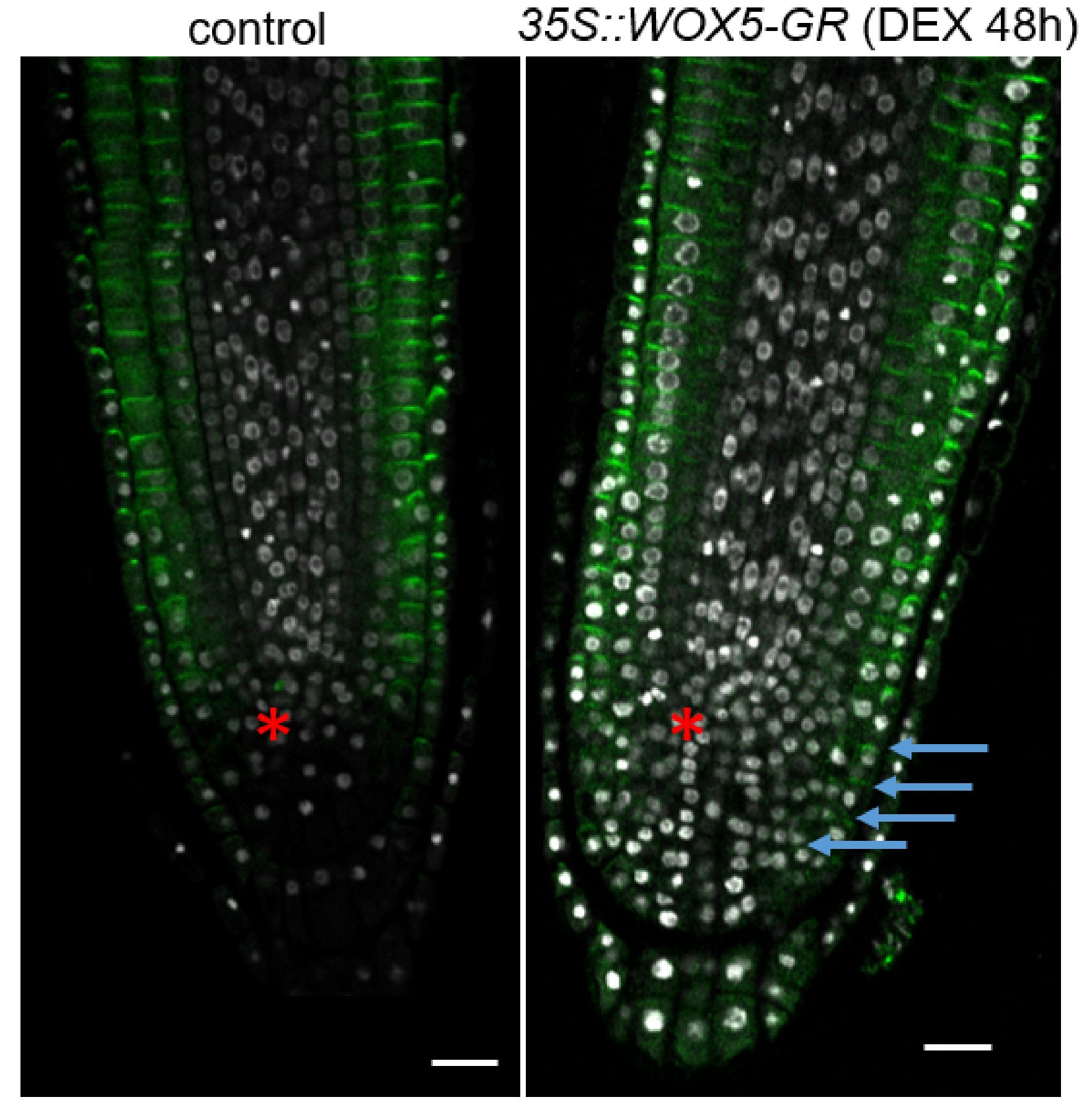

Supplement: Supplementary Figure 4 — PIN2 expression domain did not change significantly in 35S:WOX5-GR root tips after 48 h of DEX exposure, except being additionally expressed in the extra layers of the lateral root cap. Immunolocalization for PIN2 at 0 and 48 h time points of DEX exposure. DAPI is in white. The QC location is marked by the red asterisks. Blue arrows mark lateral root cap layers with PIN2 expression under the QC position. The bar scale – 20 μm. [file Image_4.JPEG]

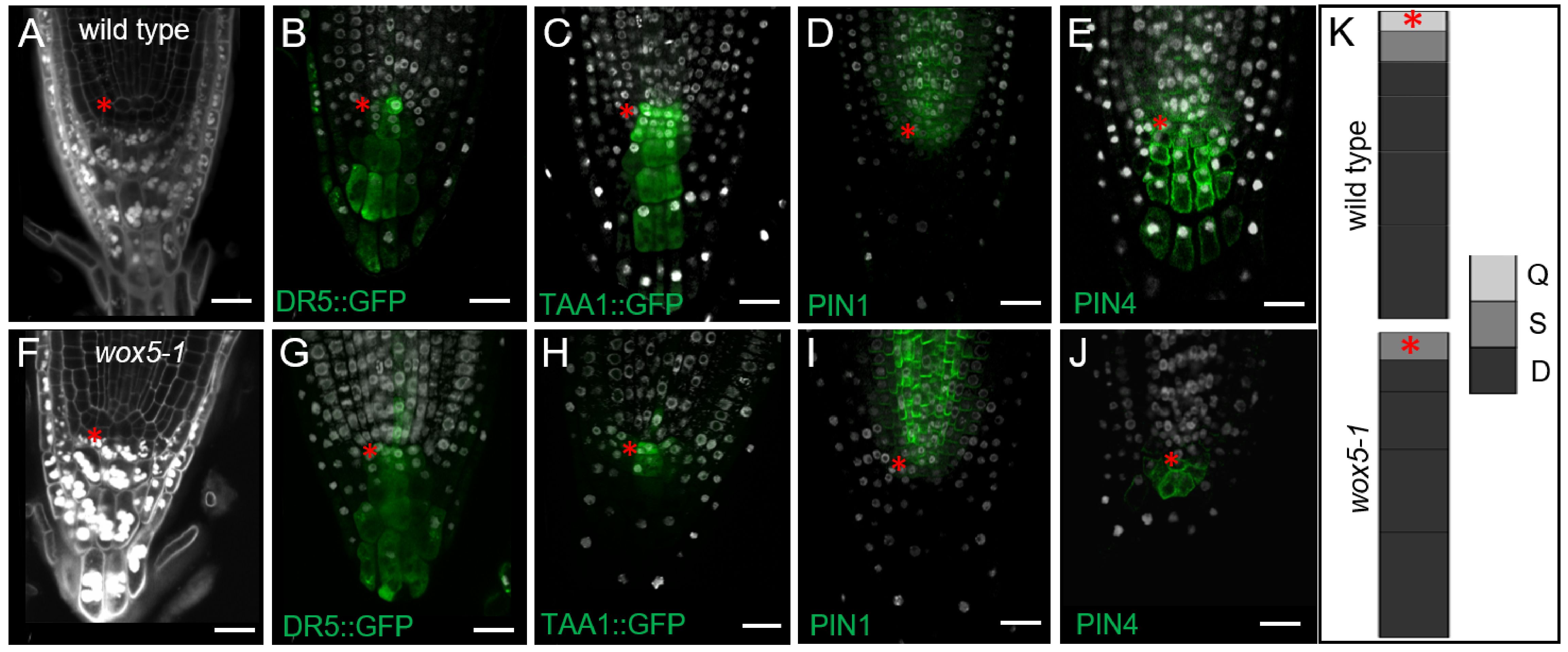

Supplement: Supplementary Figure 5 — Knockout mutant wox5-1 compared to wild type. (A) Anatomy of wild type root tip. (B) DR5:GFP expression in wild type root tip. (C) TAA1:TAA1-GFP expression in wild type root tip. (D) Immunolocalization of PIN1 in wild type root tip. (E) Immunolocalization of PIN4 in wild type root tip. (F) Anatomy of wox5-1 root tip. (G) DR5:GFP expression in wox5-1 root tip. (H) TAA1:TAA1-GFP expression in wox5-1 root tip. (I) Immunolocalization of PIN1 in wox5-1 root tip. (J) Immunolocalization of PIN4 in wox5-1 root tip. (K) Calculation results for the cell ensembles in wild type and wox5-1 root tips at 1,500 calculation steps. The gray rectangles display the cell states [Quiescent (Q), Stem (S), and Differentiation (D)]. The QC location is marked by the red asterisks. [file Image_5.JPEG]

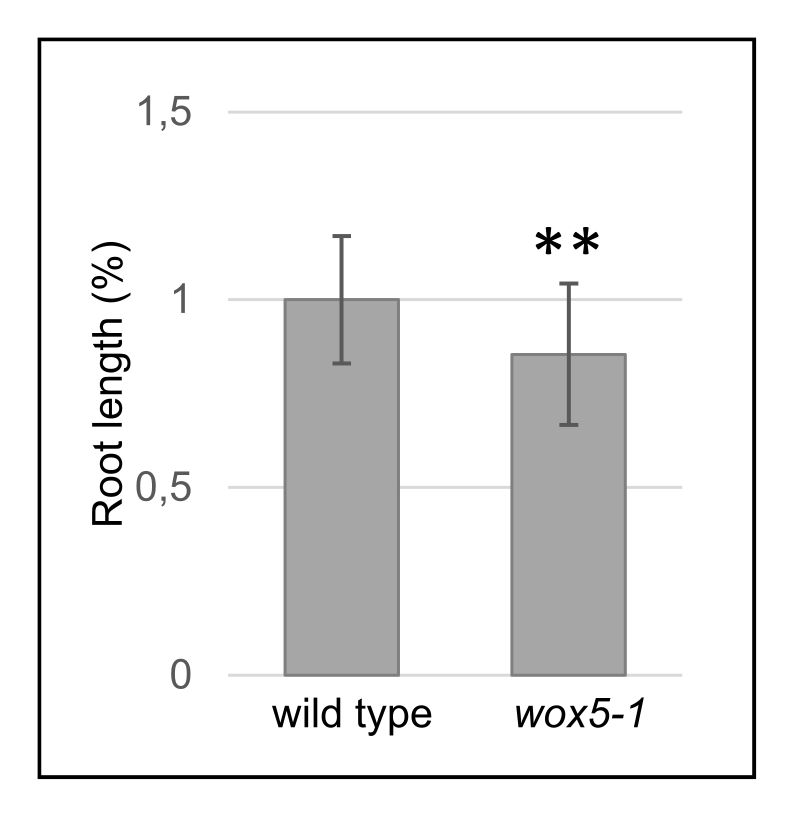

Supplement: Supplementary Figure 6 — Shortening of the wox5-1 primary root compared to the wild type. The statistical significance of the differences was estimated using Welch’s t-test (∗p < 0.05; ∗∗p < 0.01; ∗∗∗p < 0.001). [file Image_6.JPEG]

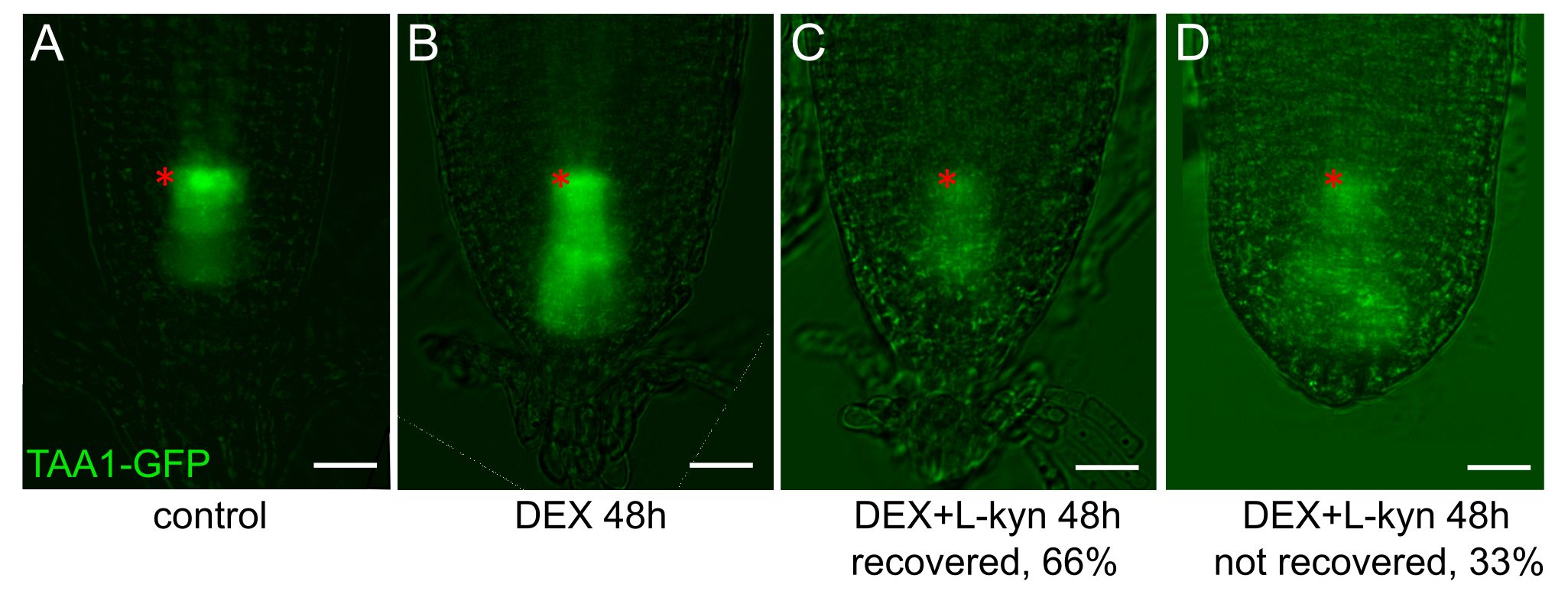

Supplement: Supplementary Figure 7 — L-Kynurenine partially rescue a portion of DEX-treated 35S:WOX5-GR TAA1:TAA1-GFP plants. (A) Mock-treated plants. (B) TAA1-GFP signal upon 48 h DEX treatment. (C) TAA1-GFP is partially rescued by L-kynurenine plants (66%, N = 20). (D) No rescue occurs in 33% of plants treated by L-kynurenine. The QC location is marked by the red asterisks. The bar scale – 20 μm. [file Image_7.JPEG]
