# Supplementary material for: Cell Dynamics in WOX5-Overexpressing Root Tips: The Impact of Local Auxin Biosynthesis
Source: Front Plant Sci. 2020 Oct 22;11:560169. doi: 10.3389/fpls.2020.560169 (PMC7642516; doi:10.3389/fpls.2020.560169)
Supplement: Supplementary Dataset 1 — Exported data from iRoCS Toolbox of three roots for Figure 2. [file Data_Sheet_1.pdf]

Supplementary Text. Mathematical model description.

2-dimensional model of auxin distribution

For modeling of auxin distribution in wild typeand the transgenic line *35S::WOX5-GR* we use two-dimensional dual-mechanism mathematical model described in Mironova et al., 2012, Hong et al., 2017. The rectangular cell layout consists of 25 rows (*i*=1…25) and 10 columns (*j*=1…10). The first and 25th rows correspond to the oldest columella and most shootward meristem cell layers, respectively. We distinguish the following four plant tissues in the layout: epidermis (*j*=1, 10), cortex (*j*=2, 9), endodermis (*j*=3, 8), stele (*j*=4, 5, 6, 7). We describe in the model the following processes: auxin synthesis, auxin degradation, diffusion and PIN-mediated active transport of auxin, and auxin dependent expression of PINs. In the model auxin moves from cell to cell directly by means of passive diffusion and active transport.

There are only three PINs in the model, PINse, PINce and PINnp. The model concentrations of auxin and PINs are calculated simultaneously. While the position of PIN expression domains is associated with auxin distribution. PINse (corresponding to PIN1 mostly, but also PIN3, PIN4 and PIN7 in the proximal expression domain) might be synthesized in stele and endodermis with rootward and lateral localization. PINce (corresponding to PIN2) might be synthesized in cortex and epidermis with rootward and lateral localization in cortex and shootward and lateral localization in epidermis. PINnp (corresponding to PIN3, PIN4, PIN7 in the distal expression domain) might be synthesized in every tissue and has non-polar localization in cells.

Here we describe the system of ODEs only for the left half (*j*=1,…,5) of the cell layout because of the radial symmetry of the root (eq. 1):

$$\begin{cases} \frac{d[PINse]_{i,j}}{dt} = K_{s,PINse}\frac{\left(\frac{[a]_{i,j}}{q_{1,PINse}}\right)^{s_{PINse}}}{1+\left(\frac{[a]_{i,j}}{q_{2,PINse}}\right)^{s_{PINse}}} - K_{d,PINse}\left(1+\left(\frac{[a]_{i,j}}{q_{3,PINse}}\right)^{h_{PINse}}\right)[PINse]_{i,j}, j = 3,4,5 \\ \frac{d[PINce]_{i,j}}{dt} = K_{s,x}\frac{\left(\frac{[a]_{i,j}}{q_{1,PINce}}\right)^{s_{PINce}}}{1+\left(\frac{[a]_{i,j}}{q_{2,PINce}}\right)^{s_{PINce}}} - K_{d,PINce}\left(1+\left(\frac{[a]_{i,j}}{q_{3,PINce}}\right)^{h_{PINce}}\right)[PINce]_{i,j}, j = 1,2 \\ \frac{d[PINnp]_{i,j}}{dt} = K_{s,PINnp}\frac{\left(\frac{[a]_{i,j}}{q_{1,PINnp}}\right)^{s_{PINnp}}}{1+\left(\frac{[a]_{i,j}}{q_{2,PINnp}}\right)^{s_{PINnp}}} - K_{d,PINnp}[PINnp]_{i,j} \\ \frac{d[a]_{i,j}}{dt} = k_\alpha(i,j) + K_{s,a} - K_{d,a}[a]_{i,j} + D \times \sum_{m\in N_{i,j}}\left([a]_m - [a]_{i,j}\right) + \sum_{x\in P} J_x(i) \end{cases}, \quad (1)$$

where $k_\alpha(i,j)$ denotes the intensity of auxin flow from the elongation zone to the meristem; $K_{s,a}$ is the auxin biosynthesis rate constant; $K_{d,a}$ is the auxin degradation rate constant; D is the auxin diffusion rate constant and $N_{i,j}$ is the neighboring cells for the cell (i,j); $J_x(i,j)$ represents the auxin flows mediated by PINs, where x denotes PINse, PINce, PINnp; $K_{s,x}$ is the synthesis rate constant; $q_{1,x}$ is the activation threshold of auxin-dependent PIN protein synthesis; $q_{2,x}$ is the saturation threshold of auxin-dependent PIN protein synthesis; s_x is the Hill coefficient, which determines the rate of PIN protein synthesis in response to changes in intracellular auxin concentration; $K_{d,x}$ is the degradation rate constant; $q_{3,x}$ is the threshold of auxin-dependent PIN protein degradation; h_x is the coefficient that defines non-linearity of auxin-regulated PIN protein degradation.

Simulation of the wild type and *35S::WOX5-GR* lines in 2-dimentional model

All parameters are taken without changes from previous simulations of auxin distribution in wild type (Hong.et al., 2017; Mironova et al., 2012) (Supplementary Table 3). To simulate the *35S::WOX5-GR* transgenic line we use the mean value of TAA1 expression changes after 24 hours of DEX incubation (Figure 3F). In accordance to this data, we increase the auxin synthesis rate constant (Supplementary Table 3). We use steady-state model solution for wild type as an initial

data for simulation of auxin distribution in the *35S::WOX5-GR* transgenic line. Auxin maximum that corresponds to the QC (Figure 5B) has the shift in the new steady-state model solution. The shift in auxin maximum location suggests an increase in the number of columella layers. At the same time simulation demonstrates an expansion of PINnp (non-polar localized transporters) expression domain with the shootward shifting of PINse and PINce expression domains without any significant differences in expression levels (Figure 5C-E). Thus we see not only the expansion in auxin-reach area and PINnp expression domain that corresponded changes in DR5::GFP, PIN1::PIN1-GFP and PIN4::PIN4-GFP expression but also the appearance of additional columella layers in the modeled root, which was caused by changes in auxin synthesis rate only.

1-dimensional dynamical model with growth, division and detachment of cells

To simulate cell behavior in wild type, the *wox5-1* mutant and the *35S::WOX5-GR* transgenic line we developed dynamical 1D computational model where we considered only distal part of the root apical meristem, namely, the QC, CSCs and all their descendants. To define events of division and detachment we use two thresholds of auxin concentration (S_{CSC} and S_D) analogously to the model described in the Dubreuil et al., 2018. However, we also introduced an additional threshold which determines the ability of cells to grow and divide (S_{QC}). Thus, we have four following auxin-dependent cell states in the model: *Quiescent* (when auxin concentration in the cell is above S_{QC}), *Stem* (when auxin concentration in the cell is between S_{CSC} and S_{QC}), *Differentiation* (when auxin concentration in the cell is between S_D and S_{CSC}), and *Detachment* (when auxin concentration in the cell is less than S_D).

The model also includes TAA1-dependent auxin biosynthesis and PIN-mediated auxin active transport. Previously it was shown that decrease in auxin response in *axr3-1* mutant having the stable form of IAA17, an auxin signal repressor, cause the four-fold increase in TAA1 expression, wider domain of WOX5 expression and reduced differentiation of CSC descendants (Tian et al., 2014). On the one hand Tian and co-authors established feedbacks between the auxin signaling pathway and the WOX5 and TAA1 gene, on the other hand, TAA1 expresses only in cells with high auxin level, altogether these facts allow us to describe the expression of TAA1 protein depending on auxin. Here in our simulation we combine TAA1-dependent auxin biosynthesis and auxin transport with growth, division and differentiation of columella cells. Active and passive transport are described as in the model with the rectangular cell layout, reported above. However, we consider only nonpolar localized PINnp. To simplify the calculations we assume that PINs concentration is in equilibrium at every moment. Such assumption allows us to exclude the PINnp variable from the calculation with preservation feedback loop between auxin and PINs. Thus, we consider the equilibrium concentration of PINnp proteins (2) for calculate auxin active transport.

$$Fpin_i = \frac{\left(\frac{a_i}{q_1}\right)^s}{1+\left(\frac{a_i}{q_2}\right)^s}, \qquad (2)$$

where q_1 is the activation threshold of auxin-dependent PIN protein synthesis; q_2 is the saturation threshold of auxin-dependent PIN protein synthesis; s is the Hill coefficient, which determines the rate of PIN protein synthesis in response to changes in intracellular auxin concentration.

Here is the system of ODEs for dynamical 1D computational model which consists of 3*N equation, where N is a number of columella cells (eq. 3-7).

$$\begin{cases} \frac{da_i}{dt} = \left(k_\alpha + k_s + k_{TAA} * TAA_i - k_d a_1 - Tpas_{out,i} - Tact_{out,i} + Tpas_{in,i} + Tact_{in,i} - a_i * \frac{dL_i}{dt}\right)/L_i \\ \frac{dTAA_i}{dt} = \left(K_s * \frac{\left(\frac{a_i}{z_1}\right)^h}{1+\left(\frac{a_i}{z_2}\right)^h} - K_d * TAA_i - L_i * \frac{dL_i}{dt}\right)/L_i \\ \frac{dL_i}{dt} = \begin{cases} 0, if\ a_i > s_{QC}\ and\ L_i \geq 1 \\ \frac{(5-L_i)}{a_i} * K_{grow} \end{cases} \end{cases} \qquad (3)$$

$$Tpas_{out,i} = \begin{cases} 2 * D * a_i + (1 - p_{lat}) * 2L_i * D * a_i, for\ i = 1, \dots, n-1 \\ D * a_i + (1 - p_{lat}) * 2L_i * D * a_i, for\ i = n \end{cases} \quad (4)$$

$$Tpas_{in,i} = \begin{cases} D * a_{i+1}, for\ i = 1 \\ D * a_{i+1} + D * a_{i-1}, for\ i = 2, \dots, n-1 \\ D * a_{i-1}, for\ i = n \end{cases} \quad (5)$$

$$Tact_{out,i} = \begin{cases} \frac{1}{2*L_i+1} * Fpin_i * a_i + (1 - p_{lat}) * \frac{2L_i}{2*L_i+1} * Fpin_i * a_i, for\ i = 1, n \\ \frac{2}{2*L_i+2} * Fpin_i * a_i + (1 - p_{lat}) * \frac{2L_i}{2*L_i+2} * Fpin_i * a_i, for\ i = 2, \dots, n-1 \end{cases} \quad (6)$$

$$Tact_{in,i} = \begin{cases} \frac{1}{2*L_{i+1}+2} * Fpin_{i+1} * a_{i+1}, for\ i = 1 \\ \frac{1}{2*L_{i-1}+1} * Fpin_{i-1} * a_{i-1} + \frac{1}{2*L_{i+1}+2} * Fpin_{i+1} * a_{i+1}, for\ i = 2 \\ \frac{1}{2*L_{i-1}+2} * Fpin_{i-1} * a_{i-1} + \frac{1}{2*L_{i+1}+2} * Fpin_{i+1} * a_{i+1}, for\ i = 3, \dots n-2 \\ \frac{1}{2*L_{i-1}+2} * Fpin_{i-1} * a_{i-1} + \frac{1}{2*L_{i+1}+1} * Fpin_{i+1} * a_{i+1}, for\ i = n-1 \\ \frac{1}{2*L_{i-1}+2} * Fpin_{i-1} * a_{i-1}, for\ i = n \end{cases} \quad (7)$$

where a_i denotes auxin concentration in cell i; TAA_i is concentration of TAA1in cell i; k_α is the intensity of auxin flow from the upper part of the root; k_s is the basal auxin synthesis rate constant; k_{TAA} is the TAA1-dependent auxin synthesis rate constant; k_d is the auxin degradation rate constant; $Tpas_{out,i}$ is the function of auxin passive transport from cell i; $Tact_{out,i}$ is the function of auxin active transport from cell i; $Tpas_{in,1}$ is the function of auxin passive transport to cell i; $Tact_{in,1}$ is the function of auxin active transport to cell i; K_s is the TAA1 synthesis rate constant; K_d is the TAA1 degradation rate constant; z_1 is the activation threshold of auxin-dependent TAA1 protein synthesis; z_2 is the saturation threshold of auxin-dependent TAA1 protein synthesis; h is the Hill coefficient, which determines the rate of TAA1 protein synthesis in response to changes in intracellular auxin concentration; L_i is length of cell i; L_{max} is maximum permitted cell length; K_{grow} is the growth rate constant; S_{QC} is the growth thresholds; D is the auxin diffusion rate constant; $Fpin_i$ is the equilibrium concentration of nonpolar PIN proteins.

Initial data and the adjustment of the model parameter values in wild type

The initial cell lengths are estimated from the experimental data (Supplementary Table 4). We use the steady state solution for not growing system consisting of six cells, as initial data on auxin and TAA1 concentrations for columella growth simulation. The model is calculated iteratively. After 100 steps of ODE calculation, we check conditions for cell division or cell detachment for all cells. Then system of ODEs is altered in accordance to the new number of columella cells. After that we start next 100 steps of ODE calculation. The condition for the division of cell *i* is the following: $S_{CSC} \le a_i < S_{QC}$ and $L_{min} \le L_i \le L_{max}$. We consider that very small cells do not have the ability to divide (Schiessl et al., 2012, Sablowski, Dornelas, 2013) and too large cells have already differentiated. The cell *i* detaches in the model when its auxin concentration falls lower than the last threshold ($a_i < S_D$).

The set of parameter values for the 1D dynamic model of auxin distribution in the columella is presented in Table S4. We adjust most of the model parameters based on the results obtained in the 2D mathematical model. We use parameter values from the 2D model for the following parameters: the degradation rate constant for auxin (k_d), the diffusion transport rate constant (D), the basal synthesis rate constant for auxin (k_s), and all parameters describing active auxin transport (q_1, q_2, s).

We adjust the value of auxin lateral flow compensation (p_{lat}) based on the steady-state solution of the 2D model for wild type (Supplementary Table 5). For this we estimate the difference between lateral auxin flux from (4; 5) cell to (4; 4) one and vice versa.

We carry out the adjustment of the remaining part of model parameters with the fixed number of cells and zero growth rate constant (K_{grow}=0).

We carry out the selection of parameter values for auxin-dependent TAA1 synthesis (z_1, z_2, h) for wild type in the way that the expression domain of TAA1 is limited to the QC and two adjacent cells (Supplementary Table 5).

The intensity of auxin flow from the upper part of the root ($k\alpha$) and the TAA1-dependent auxin synthesis rate constant (k_{TAA}) are adjusted so that the auxin level in the QC is the same as observed in the 2D model (Supplementary Table 5).

Thresholds for growth, division and detachment of cells (s_{QC}, s_{CSC}, s_D) are set so that the processes of division and detachment are consistent in time and the number of cells in the columella remains constant for wild type (Supplementary Table 5). The first cell (QC) should not have grown and divided and each CSC division is followed by detachment of the last DCC.

We take the synthesis rate constant for TAA1 (K_s) and the degradation rate constant for TAA1 (K_d) equal for wild type (Supplementary Table 5).

Simulation of the cell dynamics in columella of wild type, *wox5-1* and *35S::WOX5-GR* lines

We simulate the dynamical cell behavior in two types of roots: wild type and *35S::WOX5-GR* after the parameter adjustment. We change only the synthesis rate constant for TAA1 (K_s) taking into account the changes in the TAA1 expression level.

In the case of wild type, we observe the balance between cell division and cell detachment, so that the cell number remains constant (6 cells). As soon as the cell corresponding to the CSC divides, then after several steps the last DCC detaches (Supplementary Video 1). The first cell corresponding to the QC does not grow and divide, the second cell corresponding to the CSC divides whereas the remaining cells are only able to grow throughout the all calculation period up to 3000 steps (Supplementary Video 1). It should be noted that in the model only one cell exists in the stem state (is able to divide). In addition, the expression domain of the TAA1 and auxin distribution in the model remains stable throughout the calculation time (Figure 6B, Supplementary Video 1).

For the *35S::WOX5-GR* line we varied TAA1 synthesis rate constant (K_s) according to the confidence interval [1.24; 2.40] of TAA1 expression changes at 12 h after DEX activation (Supplementary Table 2, Figure 3). Very small changes ($1.24 \leq K_s \leq 1.39$) in TAA1 synthesis rate do not affect cell dynamics in the model. Small ($1.4 \leq K_s \leq 2$) and medium ($2.01 \leq K_s \leq 2.18$) values of the TAA1 synthesis rate led to an increase in the number of small undifferentiated cells up to one-two or three-five additional layers, respectively, with the maintenance of the balance between division and decapitation and expansion of TAA1 and PINnp expression domains (Supplementary Video 2). We called this model mode as “quasy-balansed”. Significant expansion of the TAA1 expression domain during the calculation time, up to the size of the entire columella is observed when $2.19 \leq K_s \leq 2.40$ (Figure 5E, Supplementary Video 3). At the same time, auxin concentration in the cell layout increases very strongly with time (Figure 5D). It should be noted that the model predicts an imbalance between divisions and detachment of the columella cells in this transgenic line. Namely, divisions occur much more often than the detachment of the last DCC. We observe significant increase in the number of small cells and reduction in the number of differentiated cells in the model that fits the experimental data (Figure 5C, Supplementary Video 3). We called this model mode as “broken-balance”. However, despite the large number of small cells, not all cells are able to divide, but only those located in the distal part of the columella. The pool of non-

dividing cells in the "quiescent" state is formed in the proximal part of the columella (nearby the QC), and the number of such cells increases during the calculation time.

There is a very rapid increase in the size of the columella due to the huge number of divisions with long-term calculation of the model (Supplementary Video 3). The number of the columella cells reach more than 20 cells already at the 200 step and continues to increase. Consequently, we might conclude that in the real root these divisions would continue until all the sources moving rootward are exhausted.

The model shows that taking into account WOX5-dependent modulation of the auxin synthesis enzyme TAA1 expression is sufficient to explain the experimentally observed changes in the structure of the columella in wild type and the *35S::WOX5-GR* transgenic line.

We varied TAA1 synthesis rate constant (K_s) also downward within the interval [0.50; 0.95] . We observe the decrease in columella length by one cell in average when $0.50 \leq K_s \leq 0.84$ (Supplementary Video 4). The second cell corresponding to the CSC is mostly not able to divide. The TAA1 expression domain shorten by one cell with the reducing of their expression level (Supplementary Video 4). At the same time, the auxin level in the cell layout only slightly decreases. It is important to note that the first cell corresponding to the QC is able to divide, instead of retained the quiescent state. Thus, the 1D dynamical model predicts decrease in auxin concentration level and that the QC may undergo premature and more frequent divisions due to decrease in TAA1 synthesis rate. We considered that decrease in TAA1 expression might have place in *wox5-1* knockout mutant line. We checked TAA1 and DR5 expressions into the knockout line together with anatomical structure and proved model prediction (Supplementary Figure 5F-J).

In addition, we decreased TAA1-dependent auxin synthesis rate constant (k_{TAA}) down to the 50 percent of control value for the modeled *35S::WOX5-GR* upon DEX treatment. For this purpose, we used calculated model values on the model step 1300 for the "broken-balance" mode as initial data for the new calculations. We conducted ten separated simulations for the values of the TAA1 synthesis rate constant (*Ks*) within the interval [2.19; 2.40] established for the activated "broken-balance" mode. Whatever *Ks* value was set, already after the 20 steps of the calculation or less, the cell dynamics approaches the "quasy-balansed" mode for the *35S::WOX5-GR* line, while after another 200 steps the cell dynamics becomes the same as observed for the wild type (Supplementary Video 5). Thus the model predicted that such partially inhibition of TAA1-dependent auxin synthesis in *35S::WOX5-GR* line upon DEX treatment led to the restoring of the wild type phenotype over time.

1-dimensional model parameters variation

We perform the parameters variation in the dynamic 1D model for a set of parameters corresponding to wild type. The value of only one parameter is changed at a time, each calculation is carried out up to 3000 steps, and the initial data concerning the cell size are not changed.

The study on the influence of the intensity of auxin flow from the upper root part (k_α) on the cell dynamics shows that there are several characteristic behaviors in the model. When the k_α value is in the interval [2.8; 3.2], the model maintains the cellular dynamics which is characteristic for wild type, namely, the processes of cell division and detachment are coordinated and the number of columella cells remains constant. The number of cells is also constant for the interval [2.1; 2.7], but on average they were one cell less than in the control. In this case, the QC loses the Quiescent state and starts to divide like in the mutant line *wox5-1* (Supplementary Figure 5K). A complete disappearance of the stem cell pool occurs and the number of cells decreases to two. At the same time, they grow and reach the maximum possible size by 3000 calculation steps when the k_α value decreases to less than 2.0. When the k_α value is in the interval [3.3; 5.3], the cellular dynamics is

generally like in the control, except that the number of cells in the Quiescent state corresponding to QC rises up to two or three, and the total number of cells is on average one to two more than in control. When the k_α value is more than 5.4, the Stem cell pool disappears completely, that is, the columella consists only of Quiescent cells and Differentiated cells.

The variation of the value of the TAA1-dependent synthesis rate constant for auxin (k_{TAA}) shows almost the same characteristic behavior. When k_{TAA} value is less than 2.4, the model keeps the *wox5-1* mutant behavior. The interval [2.5;11.5] corresponds to the normal cellular dynamics for control or appearance of one or two additional layers in Quiescent state. Changes of k_{TAA} value in the interval [11.5; 14.4] lead to the cell dynamics corresponding to the *35S::WOX5-GR* line characteristic behavior. Stem cell pool disappears completely when the k_{TAA} value is more than 14.5.

Ten times decrease or increase of the degradation and basal synthesis rate constants for auxin (k_d and k_s) does not lead to a change in the cellular dynamics.

Thus, the one-dimensional dynamical model of auxin distribution is the most sensitive to changes in the intensity of auxin flow from shoot (k_α) and less sensitive to changes in the rate of degradation and basal synthesis for auxin (k_d and k_s). Only changes in the TAA1-dependent synthesis rate constant for auxin (k_{TAA}) might lead to the cell dynamics that is typical for the *35S::WOX5-GR* line.

We investigate the model for the sensitivity to changes in threshold values for growth, division and detachment and maximal and minimal cell size for division. However, variations of all these parameters either has no effect on cell dynamics, or leads to a slight change in the average number of cells with maintenance of the behavior corresponded to the control.

References

Dubreuil C, Jin X, Grönlund A, Fischer U. 2018. A local auxin gradient regulates root cap self-renewal and size homeostasis. Current Biology **28**(16):2581-7.

Hong JH, Savina M, Du J, Devendran A, Ramakanth KK, Tian X, Sim WS, Mironova VV, Xu J. 2017. A sacrifice-for-survival mechanism protects root stem cell niche from chilling stress. Cell **170**(1), 102-113.

Mironova VV, Omelyanchuk NA, Novoselova ES, Doroshkov AV, Kazantsev FV, Kochetov AV, Kolchanov NA, Mjolsness E, Likhoshvai VA. 2012. Combined in silico/in vivo analysis of mechanisms providing for root apical meristem self-organization and maintenance. Annals of botany **110**(2), 349-360.

Pi L, Aichinger E, van der Graaff E, Llavata-Peris CI, Weijers D, Hennig L,Laux T. 2015. Organizer-derived WOX5 signal maintains root columella stem cells through chromatin-mediated repression of CDF4 expression. Developmental cell **33**(5), 576-588.

Sablowski R, Carnier Dornelas M. 2013. Interplay between cell growth and cell cycle in plants. Journal of Experimental Botany **65**(10), 2703-2714.

Schiessl K, Kausika S, Southam P, Bush M, Sablowski R. 2012. JAGGED controls growth anisotropy and coordination between cell size and cell cycle during plant organogenesis. Current Biology **22**(19), 1739-1746.

Tian H, Wabnik K, Niu T, Li H, Yu Q, Pollmann S, Friml J. 2014. WOX5–IAA17 feedback circuit-mediated cellular auxin response is crucial for the patterning of root stem cell niches in Arabidopsis. Molecular plant **7**(2), 277-289.

Supplementary Table 1. Quantitative analysis of mitotic and S-phase nuclei distributions in *35S::WOX5-GR* root tips at 0, 12, and 48 h of DEX incubation. Analysis performed on the root tip models created in iRoCs Toolbox (Schmidt et al., 2014) using 3D confocal images labelled by EdU/DAPI.

Hours of DEX incubation		Columella	Lateral root cap	Proximal meristem
0	Mitosis	0±1	36±14	272±41
	S-phase	17±5	181±31	469±50
12	Mitosis	6±2	44±20	218±37
	S-phase	41±10	190±28	235±42
48	Mitosis	24±5	59±13	102±24
	S-phase	69±16	192±30	134±43

Supplementary Table 2. The primers used for qRT-PCR.

Name	primers_name	primers_sequence 5'->3'
PIN1	DN_PIN1.2_F	GGTTTGGAGGAAACTTATT
	DN_PIN1.2_R	CTGAGAGTATGGAGATAGA
PIN4	DN_PIN4.2_F	GATAATGGTGTGGAGAAA
	DN_PIN4.2_R	CTGAGAGTATGGAGATGG
TAA1	dn_TAA1_F	CACTCTCTTCACTAGCC
	dn_TAA1_R	TCCCACTTGTACATACC
GFP	dn_F_GFP	CTTTCGGTTATGGTGTTC
	dn_R_GFP	GTAGTTCCCGTCATCTT
**Reference gene**		
EEFalpha4	DN5_EEFalpha4_F	CTGGAGGTTTTGAGGCTGGTAT
	DN6_EEFalpha4_R	CCAAGGGTGAAAGCAAGAAGA

Supplementary Table 3. Parameter Settings Used in the 2D Model.
cu and *tu* are concentration and time units, respectively, *dl* denotes the dimensionless parameter.

Parameter	Symbol	Units	Value wt /35S::WOX5-GR
The intensity of auxin flow from the upper root part	k_a	*cu/tu*	0.6
The degradation rate constant for PINse	$K_{d,PINse}$	*1/tu*	1000
The degradation rate constant for PINce	$K_{d,PINce}$	*1/tu*	1000
The degradation rate constant for PINnp	$K_{d,PINnp}$	*1/tu*	1000
The degradation rate constant for auxin	$K_{d,a}$	*1/tu*	0.005
The synthesis rate constant for PINse	$K_{s,PINse}$	*1/tu*	1000
The synthesis rate constant for PINce	$K_{s,PINce}$	*1/tu*	1000
The synthesis rate constant for PINnp	$K_{s,PINnp}$	*1/tu*	1000
The synthesis rate constant for auxin	$K_{s,a}$	*cu/tu*	0.002/0.0036
The lateral active transport rate constant for PINse	$K_{0,lat,PINse}$	*dl*	0.1

The lateral active transport rate constant for PINce	$K_{0,lat,PINce}$	*dl*	0.15
The basal active transport rate constant for PINse	$K_{0,bas,PINse}$	*dl*	0.9
The basal active transport rate constant for PINce	$K_{0,bas,PINce}$	*dl*	0.85
The apical active transport rate constant for PINce	$K_{0,apic,PINce}$	*dl*	0.85
The active transport rate constant for PINnp	$K_{0,PINnp}$	*dl*	1
The diffusion transport rate constant	D	*1/tu*	0.08
The activation thresholds of auxin-dependent synthesis of PINse	$q_{1,PINse}$	*1/cu*	0.3
The saturation thresholds for auxin-dependent synthesis of PINse	$q_{2,PINse}$	*1/cu*	10
The thresholds of auxin-dependent degradation of PINse	$q_{3,PINse}$	*1/cu*	1
The Hill coefficient, which determines the rate of PINse synthesis in response to changes in intracellular auxin concentration	s_{PINse}	*dl*	2
The coefficient that defines non-linearity of auxin-regulated PINse degradation	h_{PINse}	*dl*	6
The parameters of auxin-dependent synthesis and degradation for PINce	$q_{1,PINce}$	*1/cu*	0.1
	$q_{2,PINce}$	*1/cu*	0.8
	$q_{3,PINce}$	*1/cu*	0.2
	s_{PINce}	*dl*	2
	h_{PINce}	*dl*	4
The parameters of auxin-dependent synthesis and degradation for PINnp	$q_{1,PINnp}$	*1/cu*	10
	$q_{2,PINnp}$	*1/cu*	10
	s_{PINnp}	*dl*	4

Supplementary Table 4. Cell sizes for the QC and the columella cells used as initial data. The length units use.

QC	CSC	CSCD	DCC1	DCC2	DCC3
1	1.03	2.14	2.79	3.14	4.74

Supplementary Table 5. Parameter Settings Used in the 1D Model.
mu – is the mass units, *cu* – is the concentration units, *tu* – is the time units, *vu* – is the size units, *dl* – denotes the dimensionless parameter.

Parameter	Symbol	Units	Value
The intensity of auxin flow from the upper part	k_α	*mu/tu*	3.1
The degradation rate constant for auxin	k_d	*vu/tu*	0.005
The diffusion transport rate constant	D	*1/tu*	0.08
The basal synthesis rate constant for auxin	k_s	*mu/tu*	0.001
The TAA1-dependent synthesis rate constant for auxin	k_{TAA}	*vu/tu*	6
The synthesis rate constant for TAA1	K_s	*mu/tu*	1*
The degradation rate constant for TAA1	K_d	*vu/tu*	1
The activation thresholds of auxin-dependent synthesis of TAA1	z_1	*1/cu*	10
The saturation thresholds for auxin-dependent synthesis of TAA1	z_2	*1/cu*	7.8
The Hill coefficient, which determines the rate of TAA1 synthesis in response to changes in intracellular auxin concentration	h	*dl*	12
The activation thresholds of auxin-dependent synthesis of PINnp	q_1	*1/cu*	10
The saturation thresholds for auxin-dependent synthesis of PINnp	q_2	*1/cu*	10
The Hill coefficient, which determines the rate of PINnp synthesis in response to changes in intracellular auxin concentration	s	*dl*	4
The growth rate constant	K_{grow}	*dl*	0.0001
The parameter of auxin lateral flow compensation	p_{lat}	*dl*	0.9
Maximal allowed cell size	L_{max}	*vu*	5
Minimal cell size for division	L_{div1}	*vu*	1.5
Maximal cell size for division	L_{div2}	*vu*	2.5
Threshold for cell growth	s_{QC}	*cu*	12.7
Threshold for cell division	s_{CSC}	*cu*	10.5
Threshold for cell detachment	s_D	*cu*	1.5

* Value for wt; for 35S::WOX5-GR the value is varied within the confidence interval [1.24; 2.40] of TAA1 expression changes observed experimentally.

Legends of the Supplementary Videos 1-5

Supplementary Video 1. Simulation of cell dynamics in the root tip of wild type plants. Blue and green plots reflect the auxin and TAA1 concentration in the cells respectively. X-axis indicates cell length (QC length in wild type (wt) is taken as 1). Y-axis indicates auxin concentration in concentration units. Set of gray rectangles displays cells in different cell state shown by color intensity: Quiescent state has the lowest intensity, Stem state has the middle one, Differentiation state has the highest one. Red lines depict three model thresholds.

Supplementary Video 2. Simulation of cell dynamics in the root tip of *35S::WOX5-GR* plants under DEX induction (*quasi-balance* mode). Blue and green plots reflect the auxin and TAA1 concentration in the cells respectively. X-axis indicates cell length (QC length in wild type (wt) is taken as 1). Y-axis indicates auxin concentration in concentration units. Set of gray rectangles displays cells in different cell state shown by color intensity: Quiescent state has the lowest intensity, Stem state has the middle one, Differentiation state has the highest one. Red lines depict three model thresholds.

Supplementary Video 3. Simulation of cell dynamics in the root tip of *35S::WOX5-GR* plants under DEX induction (*broken-balance* mode). Blue and green plots reflect the auxin and TAA1 concentration in the cells respectively. X-axis indicates cell length (QC length in wild type (wt) is taken as 1). Y-axis indicates auxin concentration in concentration units. Set of gray rectangles displays cells in different cell state shown by color intensity: Quiescent state has the lowest intensity, Stem state has the middle one, Differentiation state has the highest one. Red lines depict three model thresholds.

Supplementary Video 4. Simulation of cell dynamics in the root tip of *wox5-1* plants. Blue and green plots reflect the auxin and TAA1 concentration in the cells respectively. X-axis indicates cell length (QC length in wild type (wt) is taken as 1). Y-axis indicates auxin concentration in concentration units. Set of gray rectangles displays cells in different cell state shown by color intensity: Quiescent state has the lowest intensity, Stem state has the middle one, Differentiation state has the highest one. Red lines depict three model thresholds.

Supplementary Video 5. Simulation of cell dynamics under partially inhibition of TAA1-dependent auxin synthesis in *35S::WOX5-GR* plants. Blue and green plots reflect the auxin and TAA1 concentration in the cells respectively. X-axis indicates cell length (QC length in wild type (wt) is taken as 1). Y-axis indicates auxin concentration in concentration units. Set of gray rectangles displays cells in different cell state shown by color intensity: Quiescent state has the lowest intensity, Stem state has the middle one, Differentiation state has the highest one. Red lines depict three model thresholds.
